# An mRNA-encoded scFv antibody targeting the helix-α3 of HPV18 E7 oncoprotein as a novel antiviral strategy

**DOI:** 10.1128/mbio.02627-25

**Published:** 2026-02-02

**Authors:** Feng Han, Xin-ying Guo, Ling-yan Cui, Meng-xuan Zhang, Ya-rong Zeng, Gui-qiang Wang, Jin-jin Li, Xin Chi, Ming-xia Jiang, Yue-ting Xiong, Li-zhi Zhou, Qing-bing Zheng, Hai Yu, Jun Zhang, Ting-ting Li, Ying Gu, Ning-shao Xia, Shaowei Li

**Affiliations:** 1 State Key Laboratory of Vaccines for Infectious Diseases, Xiang An Biomedicine Laboratory, School of Life Sciences, Xiamen University 199198, Xiamen, China; 2 State Key Laboratory of Molecular Vaccinology and Molecular Diagnostics, National Innovation Platform for Industry-Education Integration in Vaccine Research, NMPA Key Laboratory for Research and Evaluation of Infectious Disease Diagnostic Technology, National Institute of Diagnostics and Vaccine Development in Infectious Diseases, Xiamen University 12466, Xiamen, China; Okayama University, Kurashiki, Okayama, Japan

**Keywords:** HPV, scFv, structure, mRNA, tumor

## Abstract

Despite the availability of prophylactic vaccines, human papillomavirus (HPV) infection remains the leading viral cause of cancer worldwide. The HPV E7 oncoprotein is a key factor in cancer progression by degrading host tumor suppressor proteins, thus offering a promising target for antiviral therapy. In this study, we screened a panel of high-affinity antibodies against HPV18 E7. Through evaluation of their cytotoxic effects in HPV18-positive HeLa cells, incorporating the structure of antibodies and the structural simulation of complexes, we identified the α3 helix of the HPV18 E7 protein and its adjacent groove as a novel and effective antiviral epitope. The candidate antibody 17F2 showed efficacy in inhibiting cell proliferation and tumor formation when transfected into HeLa cells as a single-chain variable fragment (scFv). To directly assess the efficacy of the antibody and enhance the accessibility of antibody drugs, we employed an mRNA platform for scFv delivery. This approach significantly inhibited the growth of HPV18-positive tumors in the immunodeficient mouse models. Our study identifies the α3 helix of HPV18 E7 as a viable antiviral target and provides proof of concept for mRNA-encoded scFv antibodies as a novel and effective strategy to neutralize viral oncoproteins in the treatment of HPV-related cancers.

**IMPORTANCE:**

The development of effective therapeutics against human papillomavirus (HPV)-related cancers remains an urgent medical priority. While therapeutic vaccines depend on the host immune response, their efficacy can be limited in immunocompromised individuals. In contrast, antibody-based therapies that directly target viral oncoproteins represent a promising alternative with a more immediate mechanism. In this study, we identified and characterized a potent therapeutic antibody against HPV18 E7 and uncovered an unrecognized targeting epitope within this viral oncoprotein. Moreover, we addressed a major limitation of conventional antibody therapies—their inability to efficiently target intracellular proteins—by employing an mRNA-lipid nanoparticle delivery platform for intracellular expression of the antibody. As a result, we have developed a novel candidate drug with a clear mechanism, offering a new strategy for the treatment of cancers associated with HPV18.

## INTRODUCTION

Human papillomavirus (HPV) was initially isolated from plantar warts in 1975 (1). As of January 2026, the International HPV Reference Center has reported 231 distinct HPV types. HPV16 and HPV18 are responsible for 70% of cervical cancer cases worldwide (2), with HPV18-positive tumors exhibiting a more invasive phenotype and posing greater survival challenges for patients (3). In 2022 alone, an estimated 662,000 women were diagnosed with cervical cancer, and approximately 349,000 women died from the disease (4).

HPV primarily targets epithelial cells. After entry, the virus expresses early proteins, notably E6 and E7, which facilitate genome replication and promote cellular proliferation. As the infected cells differentiate, late proteins L1 and L2 are expressed, resulting in virion assembly and release (5). The sustained overexpression of E6 and E7 play the central role in oncogenesis and also represents a specific therapeutic target for HPV-induced malignancies (5).

Currently available prophylactic HPV vaccines—six of which are licensed—primarily function by eliciting neutralizing antibodies against the L1 capsid protein (6–9), effectively preventing infection but offering no therapeutic benefit for individuals already infected with HPV. A recent World Health Organization document has emphasized the need for developing therapeutic HPV vaccines (TxVs). It also highlights that cervical cancer progresses more rapidly in women living with HIV, with disease advancement potentially occurring within 5–10 years. HIV infection is associated with a six-fold increased risk of cervical cancer, delayed viral clearance, and a five-fold higher recurrence rate compared to HIV-negative women (10). TxVs aim to elicit robust antitumor immune responses by introducing endogenous oncogenic HPV proteins (11–15). Several preclinical candidates have shown the capacity to clear persistent high-risk HPV infections and induce regression of precancerous lesions (16). However, the efficacy of therapeutic vaccines is highly dependent on the host’s immune response, and their effectiveness could be significantly diminished in immunocompromised populations. This highlights the urgent need to develop novel therapeutic strategies for these individuals.

Antibody therapeutics offer an alternative approach by achieving target-specific cytotoxic effects with minimal reliance on host immunity. Despite extensive research on antibodies targeting HPV16 E7, the precise epitopes of these antibodies have not yet been identified. The high flexibility of the E7 full-length protein may hinder the formation of stable antigen-antibody complexes. Determining the epitopes of these antibodies could clarify their mechanisms of action and facilitate the selection of the most effective candidates for targeted antibody therapy.

Besides, targeting intracellular proteins such as E7 presents additional challenges, including cellular uptake, endosomal escape, and proper intracellular localization (17). While protein-based antibody delivery methods—such as sonoporation, cell-penetrating peptides, and liposomal carriers—have been explored (18, 19), clinical translation remains difficult. Alternatively, nucleic acid-based delivery allows for intracellular antibody expression; this expression utilizes retroviral vectors (20, 21). Retroviral vectors have been employed to express intracellular antibodies targeting HPV E6 and E7, demonstrating efficacy in cellular (22–24); however, it has also raised concerns about the integration of genes (25). Modified adeno-associated viruses (AAVs) serve as another major class of delivery vectors; nonetheless, AAV therapies can trigger anti-vector immunity, limiting the potential for repeat administration or future AAV-based treatments targeting different pathogens or strains (26).

Given these limitations, we aimed to develop an antiviral strategy that directly targets the HPV18 E7 oncoprotein, a critical viral driver of carcinogenesis. Our initial step involved identifying a high-affinity monoclonal antibody (17F2) that recognizes a novel conformational epitope on E7, characterized through a combination of crystallography and bioinformatics. The potent anti-oncogenic activity of 17F2, mediated through inhibition of E7’s proliferative functions, was confirmed using antibody-mediated inhibition of cell proliferative activity (AIA) and validated across cellular and animal models. To enable efficient intracellular delivery of this therapeutic agent, we engineered an mRNA-encoded single-chain variable fragment (scFv) version of 17F2. This mRNA therapeutic effectively neutralized the viral oncoprotein in HPV18-transformed cells and demonstrated significant efficacy in suppressing tumor growth *in vivo*, highlighting its potential as a novel antiviral biologic.

## RESULTS

### Identification of HPV18 E7-targeting antibodies with antiviral activity

**Fig 1:**
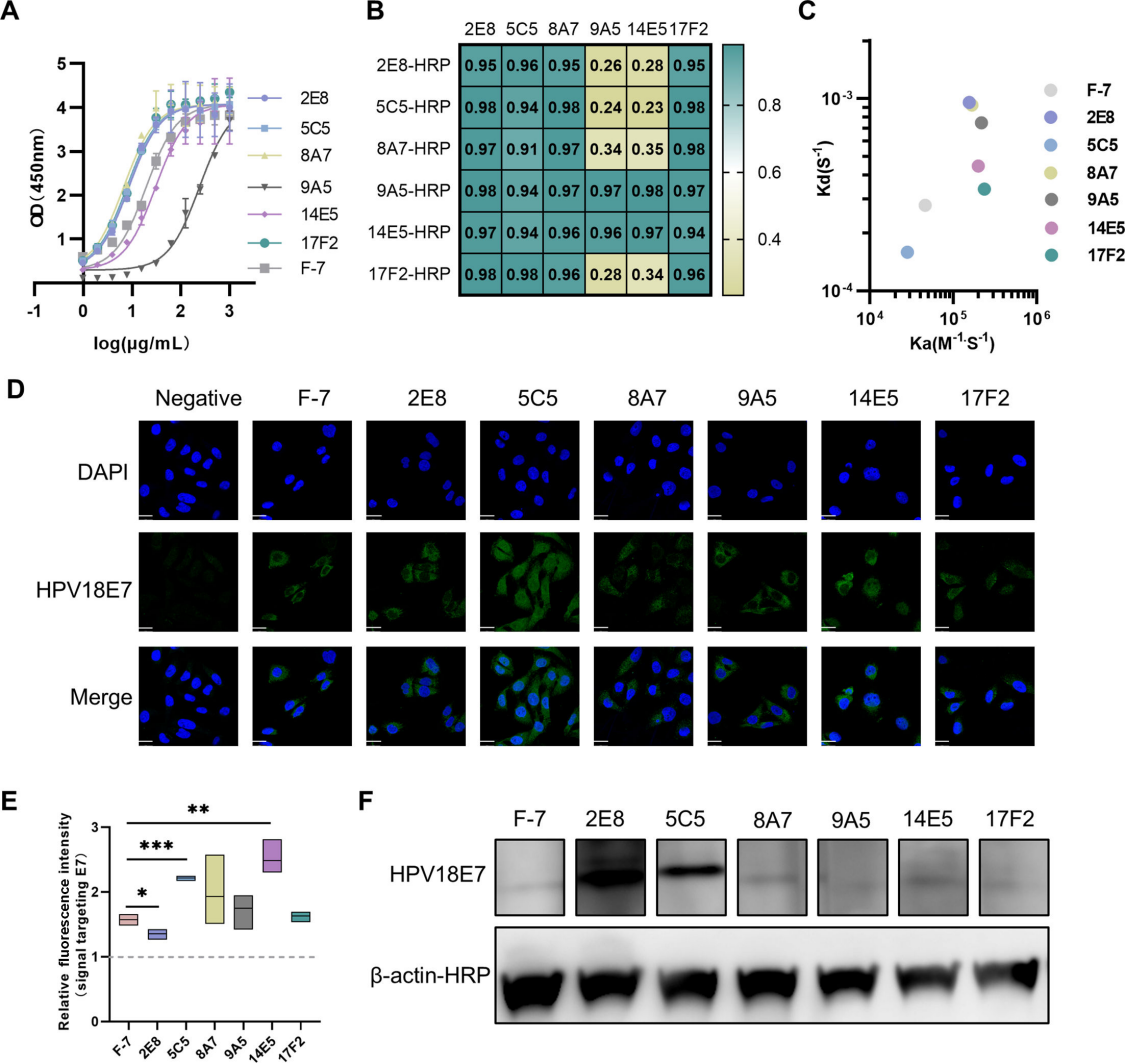
Characterization of EC₅₀ and binding properties of six anti-HPV18E7 mAbs. (**A**) Antibody against HPV18E7 binding ability analysis by ELISA. Recombinant HPV18 E7 protein (1 μg/mL) was coated onto plates. Commercial antibody F-7 as a positive control. (**B**) Competition matrix of six anti-E7 mAbs. Yellow box and black lines indicate mAbs that compete with one another. The value contained in each box is 1 − a given mAb/control across duplicate; a value <0.5 was taken to be negative binding of the detection mAb. Non-competing pairs are shown in green. (**C**) Kinetic parameters for the binding of each antibody to HPV18E7, as determined by surface plasmon resonance analysis. (**D**) Six Anti-E7 mAbs could recognize HPV18 E7 protein in the HPV18-positive HeLa cells by immunofluorescence. Scale bar = 20 um. Commercial antibody F-7 as a positive control. No primary antibody group as a negative control. The rectangular artifacts on the right side of F-7, 14E5, and 17F2 are the result of a technical issue that occurred during file conversion. (**E**) Quantitative analysis of panel **D**. The signal targeting E7 (the green signal in panel D) was quantitatively analyzed using ImageJ and normalized. Y normalization = mean density (experimental group)/average of mean density (negative control). A pairwise difference analysis was performed between the experimental and control groups using *t*-test, *n* = 3, *P < 0.05, **P < 0.01, and ***P < 0.001. (**F**) Western blot showed that Anti-E7 mAbs could recognize endogenously expressed HPV18 E7 protein in the HPV18-positive HeLa cells. β-actin as the control. This figure represents spliced gel segments combined for imaging.

We previously generated a panel of mouse monoclonal antibodies against HPV18 E7 using the hybridoma method and identified six candidates with half-maximal effective concentration (EC₅₀) values ranging from 0 to 300 ng/mL, as determined by enzyme-linked immunosorbent assay (ELISA; Fig. 1A; Fig. S1A). Among these, four clones—8A7, 17F2, 5C5, and 2E8—exhibited EC₅₀ values below 10 ng/mL, indicating superior binding performance compared to the commercial antibody F-7. Epitope competition assays revealed that clones 9A5 and 14E5, which had lower overall binding activity, recognized distinct, non-overlapping epitopes compared to the other four antibodies (Fig. 1B).

To further characterize the binding properties, we performed surface plasmon resonance (SPR) analysis for the six antibodies and F-7 (Fig. 1C; Fig. S1B). All seven antibodies displayed nanomolar-level binding affinities and a kinetic profile characterized by rapid association and slow dissociation (Fig. S1B). Interestingly, 9A5 and 14E5 demonstrated slightly higher binding affinities compared to the other four clones. This, coupled with their unique epitope recognition, suggests a potential dependency on conformation. Immunofluorescence assays confirmed that all six antibodies could recognize endogenous E7 protein in HPV18-positive HeLa cells (Fig. 1D). The quantitative analysis results indicated that the fluorescence signals targeting E7, detected by 5C5 and 14E5, were significantly stronger than those of the commercial antibody F-7, and the signals of 8A7, 9A5, and 17F2 were comparable to those of F-7 (Fig. 1E). The Western blot (WB) results also demonstrated that all six antibodies could detect E7; notably, 2E8 and 5C5 exhibited strong linear epitope recognition, as evidenced by their robust signal intensities (Fig. 1F).

**Fig 2:**
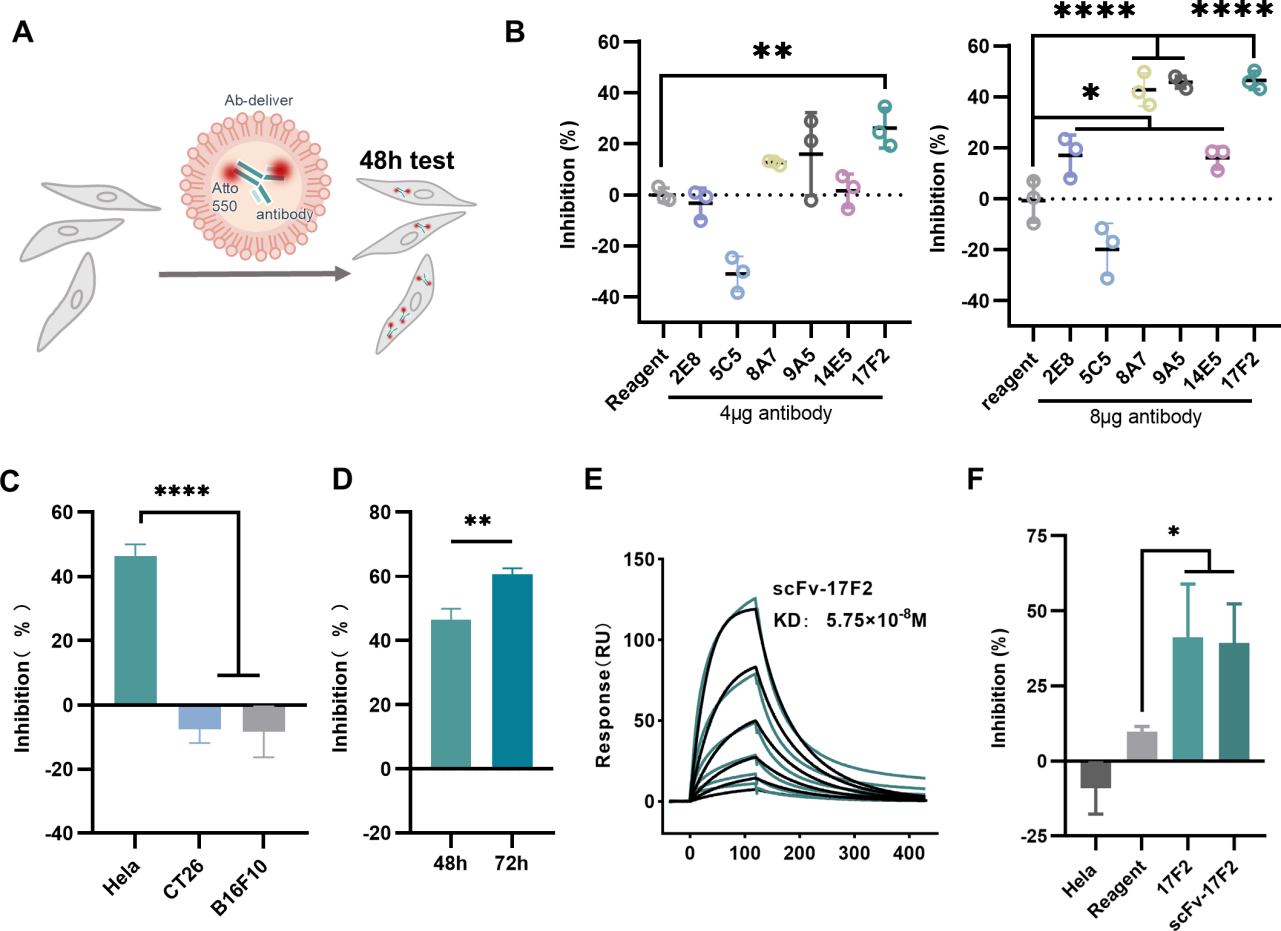
Evaluation of the targeted cytotoxicity of antibodies and scFv. (**A**) Evaluation of AIA. Fluorescently labeled antibodies entered the cell by reagent transfection, and cell proliferation activity was detected using the CCK-8 assay. (**B**) AIA of different dosages of antibodies, the reagent group as negative control. After 24 h of cell seeding, the antibodies were transfected with 4 μg (left figure) or 8 μg (right figure) per well. CCK-8 assay and inhibition rate calculation were performed 48 h after transfection. Inhibition rate = (1 − antibody OD450/average value of the control group) × 100%. (**C**) AIA of antibody 17F2 in different cell lines. After 24 h of cell seeding, the antibodies were transfected with 8 μg per well. CCK-8 assay and inhibition rate calculation were performed 48 h after transfection. (**D**) AIA of antibody 17F2 at different times. After 24 h of cell seeding, the antibodies were transfected with 8 μg per well. CCK-8 assay and inhibition rate calculation were performed 48 or 72 h after transfection. (**E**) SPR sensorgrams of scFv-17F2 binding to HPV18E7. Affinity values are indicated in the upper right corner. The black lines indicate the curve fit, while green lines indicate the measured response. (**F**) AIA of 17F2 and scFv-17F2 at equal doses. Difference analysis uses one-way ANOVA with the Dunnett’s multiple comparisons test, compared with the control group, *n* = 3, *P < 0.05, **P < 0.01, ***P < 0.001, and ****P < 0.0001. Error bars represent standard deviation.

We explored the therapeutic potential of antibodies through antibody-mediated inhibition of cell proliferative activity (AIA) assay (Fig. 2A). Antibodies were labeled with Atto-550 and transfected into HeLa cells using Ab-deliver reagent. Fluorescence microscopy confirmed efficient cellular internalization and punctate intracellular distribution; IgG-FITC served as a positive control (Fig. S2A). At 48 h post-transfection, cell proliferation assays were conducted. At a dose of 4 μg per well, 17F2 significantly inhibited HeLa cell proliferation. At 8 μg per well, enhanced inhibitory effects were observed for all antibodies except 5C5 (Fig. 2B). The proliferation-promoting effect observed with 5C5 may be attributed to non-specific protein introduction, which might be digested by tumor cells as an additional source of nutrients (27, 28).

Given its superior efficacy at lower concentrations, 17F2 was further evaluated for cell specificity. When delivered into HPV-unrelated cancer cell lines CT26 and B16F10, 17F2 exhibited no cytotoxic effects; on the contrary, it promoted proliferation, in contrast to its inhibitory effect on HeLa cells (Fig. 2C). Similar proliferation-promoting effects were observed for other antibodies in non-HPV cell lines (Fig. S2B), further supporting the hypothesis that 5C5’s proliferative effect may stem from non-specific responses. Time-course analysis revealed that the inhibitory effect of 17F2 on HeLa cells was more pronounced at 72 h post-transfection than at 48 h (Fig. 2D).

To establish a platform for rapid therapeutic antibody development, we engineered 17F2 into a scFv. The scFv-17F2 was successfully expressed and purified *in vitro* (Fig. S2C), exhibiting an affinity of 5.75 × 10⁻⁸ M for the E7 protein (Fig. 2E). AIA confirmed that scFv-17F2 retained inhibitory efficacy comparable to the full-length antibody at equivalent doses (Fig. 2F).

### The helix-α3 epitope is a novel antiviral target on the HPV18 E7 oncoprotein

To investigate the varying efficacies among the six anti-E7 antibodies, we attempted to form complexes between each antibody and recombinant HPV18 E7 protein (data not shown). Due to instability in complex formation, only the 17F2 Fab–E7 complex yielded crystals; however, density for E7 was not observed (Fig. S3A through C; Table S1). During the preparation process, we discovered that four Fab molecules were necessary to neutralize a single E7 molecule, which may drive the self-crystallization of Fab. Nonetheless, we speculate that these Fabs adopt conformations more amenable to E7 binding than individual Fab fragments.

**Fig 3:**
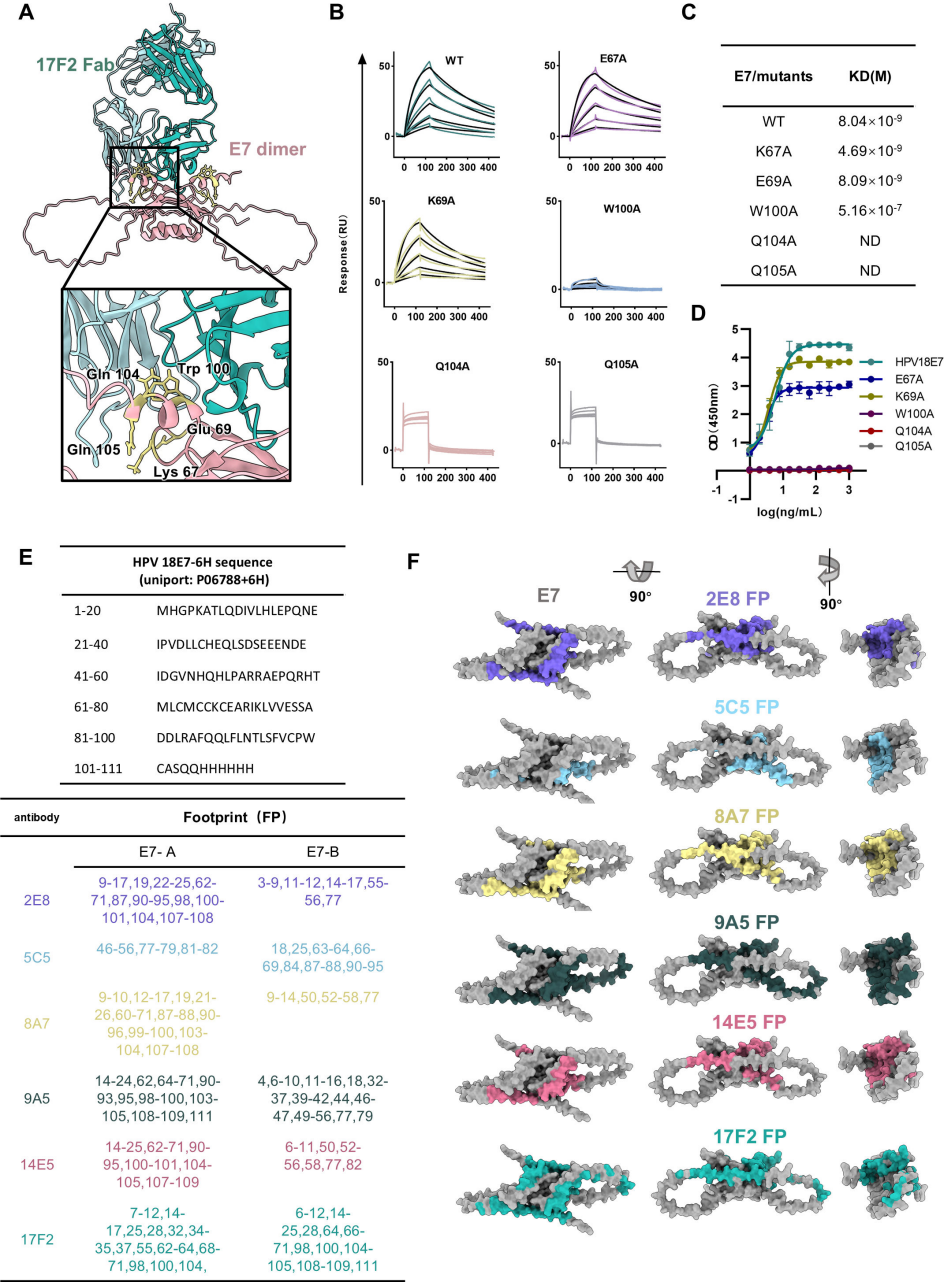
Identification of antiviral epitopes in E7. (**A**) Molecular docking of 17F2 Fab with the predicted structure of HPV18 E7. The close-up view indicates potential interaction sites between the two. (**B**) SPR sensorgrams of HPV18E7 mutants binding to 17F2. The black lines indicate the curve fit, while colored lines indicate the measured response. (**C**) Affinity values of 17F2 with HPV18 E7 mutants. (**D**) ELISA of 17F2 binding to HPV18E7 mutants. (**E**) Amino acids included in the antibody footprint. The upper part of the diagram represents the amino acids of E7 and their corresponding sequence numbers, while the lower part shows the amino acids included in the footprints of six antibodies. (**F**) Footprints of six antibodies.

Consequently, we conducted molecular docking of the Fab with the E7 dimer predicted by AlphaFold 3 (AF3) (29) (Fig. 3A). The α-helix at the C-terminal (including W100, Q104, and Q105) of E7 or the loop between the two β-sheets (including K67, E69) was predicted as the potential interaction site to 17F2 Fab. Subsequently, we expressed five E7 mutations (W100A, Q104A, Q105A, K67A, and E69A) *in vitro* (Fig. S3D and E), followed by affinity and binding evaluation with 17F2 (Fig. 3B through D). The results showed that the W100A mutant exhibited a two-order magnitude decrease in affinity compared to the wild type (WT), while Q104A and Q105A showed almost no detectable affinity with 17F2, indicating that the antibody 17F2 targets the helix-α3 at the C-terminus of E7.

As stable complexes could not be obtained for the remaining five antibodies, we determined their amino acid sequences via mass spectrometry and predicted their interactions with the E7 dimer using AF3 and Docking (Fig. S4). To confirm the prediction’s accuracy, we conducted a structural prediction of 17F2, revealing that the predicted structure closely resembles the native conformation (Fig. S5). Using PDBePISA to predict the antibody footprint (30), we combined the AF3 and molecular docking antibody footprints to create a comprehensive footprint map (Fig. 3E and F). Our findings indicate that the 17F2 Fab footprint likely spans the top of the E7 dimer, interacting with the α3 domains of both E7 monomers, which supports the exceptional stability of the 17F2 Fab–E7 complex. Conversely, 5C5 interacts with the sides and bottom of the E7 dimer, entirely avoiding the zinc finger domain at the top. The antibodies 2E8, 8A7, 9A5, and 14E5 predominantly target the grooves at the top and sides of the E7 monomer. As a result, a higher dosage is required to achieve therapeutic effects comparable to or lower than those of 17F2 for saturating and blocking the E7 dimer top. These findings highlight the significance of the helix-α3 region of E7 as a vital target for antiviral intervention.

### Intracellular expression of scFv-17F2 neutralizes E7 oncogenic activity and inhibits proliferation

**Fig 4:**
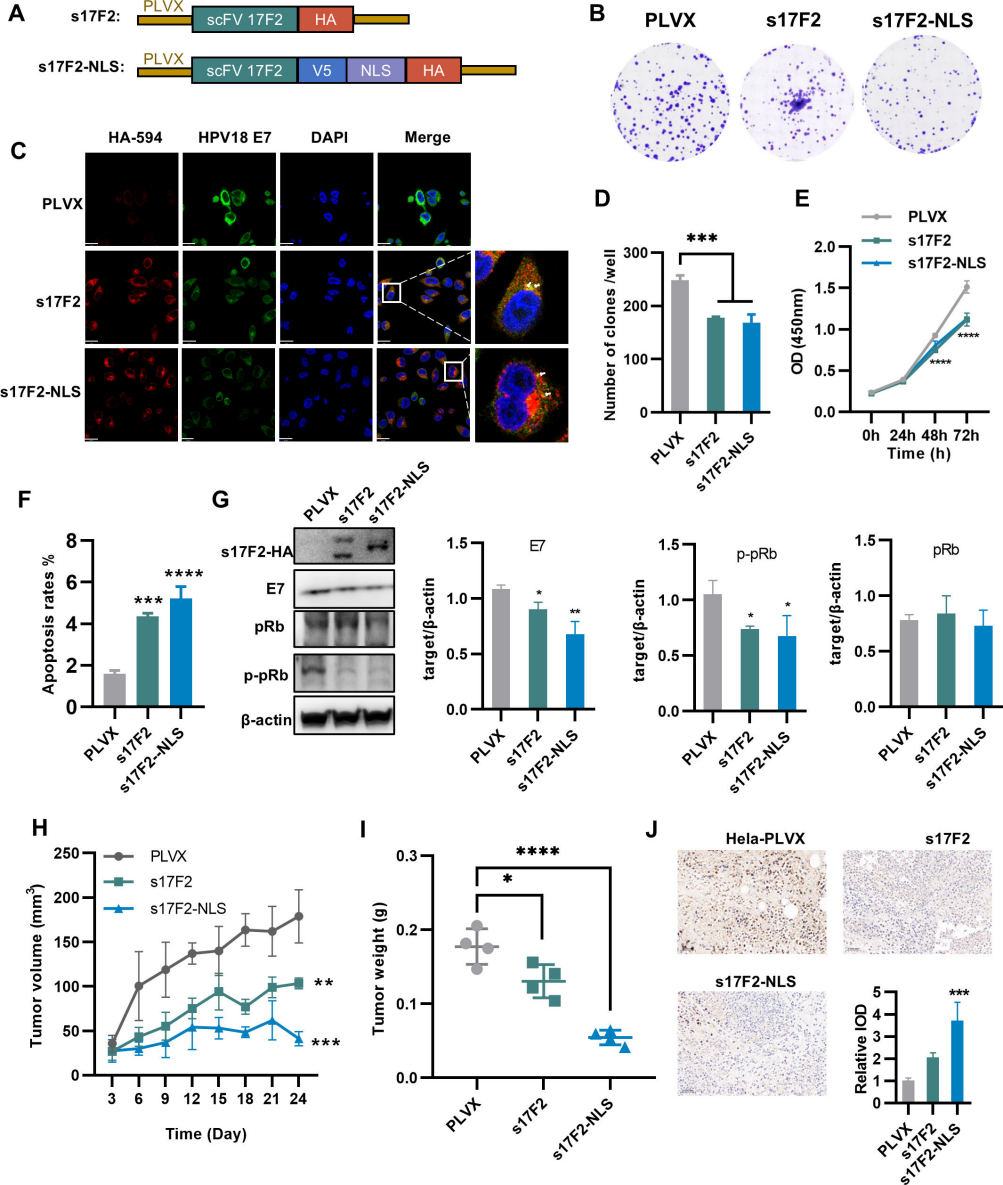
scFv-17F2 inhibits the proliferation and tumorigenicity of HPV18-transformed HeLa cells by neutralizing the E7 oncoprotein. (**A**) Schematic representation of PLVX-s17F2 and PLVX-s17F2-NLS plasmid. The V5-tag and HA-tag for immunological detection, and the NLS, are shown. (**B**) Colony formation assay. Cells were seeded with 400 cells in the six-well plates, with cell counts observed at day 10, followed by Giemsa staining. (**C**) Confocal imaging of s17F2 (red) colocalized with HPV18 E7 (green) in HPV18 positive HeLa cells, nuclei are displayed in blue. Scale bar = 20 um. The close-up shows partial co-localization. The rectangular artifacts on the right side of s17F2 are the result of a technical issue that occurred during file conversion. (**D**) Quantitative analysis of colony formation assay. (**E**) CCK-8 test. Cell proliferation activity was assessed at 0 h, 24 h, 48 h, and 72 h post-seeding, with statistical significance analyzed across the time points. Difference analysis uses one-way ANOVA with the Dunnett’s multiple comparisons test, compared with the control group (PLVX), *n* = 4, ****P < 0.0001. (**F**) Quantitative analysis of apoptosis rates in HeLa cells expressing different plasmids by flow cytometry. (**G**) Expression of different plasmids in HeLa cells induced a reduction of cellular E7 and pRb. β-actin was used as loading controls. Quantitative analysis using ImageJ. Difference analysis uses one-way ANOVA with the Dunnett’s multiple comparisons test, compared with the control group (PLVX), *n* = 3, *P < 0.05, **P < 0.01, ***P < 0.001, and ****P< 0.0001. (**H**) Tumor growth curves in mice following administration of stably transfected HeLa cells with s17F2 and s17F2-NLS. s17F2-NLS is more effective than s17F2. (**I**) The tumor weight at the end of treatment. The tumor weight in the treatment group was significantly lower than that in the control group, and the s17F2-NLS group was more effective than the S17F2 group. (**J**) Immunohistochemical and quantitative analysis of the Ki67 marker in tumor tissue. Quantitative analysis using ImageJ. Difference analysis uses one-way ANOVA with the Dunnett’s multiple comparisons test, compared with the control group (PLVX), *n* = 3, *P < 0.05, **P < 0.01, ***P < 0.001, and ****P < 0.0001.

Numerous studies have demonstrated that intracellularly expressed antibodies exhibit anti-proliferative activity on target cells (31–34). To evaluate this strategy for HPV18, we cloned scFv-17F2 into a lentiviral expression vector with an HA tag, denoted as s17F2. Based on a prior report by Amici et al. (22), which showed enhanced intracellular antibody efficacy upon addition of a nuclear localization signal (NLS), we constructed a second version incorporating NLS, designated an s17F2-NLS (Fig. 4A).

HeLa cells were transduced with either s17F2 or s17F2-NLS using the PLVX lentiviral system. After 1 week, colony formation assays revealed significant inhibition of cell proliferation in both groups (Fig. 4B and D), consistent with results from the Cell Counting Kit-8 (CCK-8) assay (Fig. 4E). Immunofluorescence staining showed co-localization of intracellularly expressed scFv with E7, while s17F2-NLS exhibited stronger perinuclear accumulation (Fig. 4C). Annexin V-PE/FITC staining revealed a marked increase in apoptosis in both groups, with s17F2-NLS demonstrating a more pronounced effect (Fig. 4F; Fig. S6).

WB analysis initially confirmed the expression of the scFv. For s17F2, we observed two bands; the larger band might be the result of incomplete cleavage of the signal peptide. For s17F2-NLS, the addition of the V5 tag and the NLS may have facilitated the complete cleavage of the signal peptide. WB further showed significant downregulation of E7 and its critical target, phosphorylated retinoblastoma protein (p-pRb), in both s17F2-expressing cell lines, while total pRb levels were maintained. No major differences were observed between s17F2 and s17F2-NLS in terms of molecular signaling (Fig. 4G). These findings support that scFv-17F2 effectively disrupts E7-mediated pRb inactivation, thereby suppressing abnormal cell proliferation.

We next assessed the *in vivo* tumor-suppressive activity of s17F2 and s17F2-NLS using a nude mouse xenograft model with stably transduced HeLa cells. Tumor volumes were measured every 3 days, and tumors were harvested and weighed on day 24. Ki67 immunohistochemistry was used to assess proliferation in excised tumors. Compared to PLVX controls, both s17F2 and s17F2-NLS significantly reduced tumor volume and weight, with lower Ki67 expression levels observed. Notably, s17F2-NLS had a more pronounced inhibitory effect than s17F2 (Fig. 4H through J).

### mRNA-mediated delivery of scFv-17F2 induces potent cytotoxicity in HPV18-transformed cells

To further evaluate the therapeutic potential of mRNA-encoded scFv antibodies, we examined the cytotoxic effects of mRNA-17F2 in HeLa cells. The *in vitro*-transcribed mRNA constructs included a poly-A tail and were capped during synthesis, resulting in two mRNA variants: m-s17F2 and m-s17F2-NLS (Fig. S7A). These mRNAs were transfected into HeLa cells using Lipofectamine 3000 to assess intracellular expression and functional outcomes.

**Fig 5:**
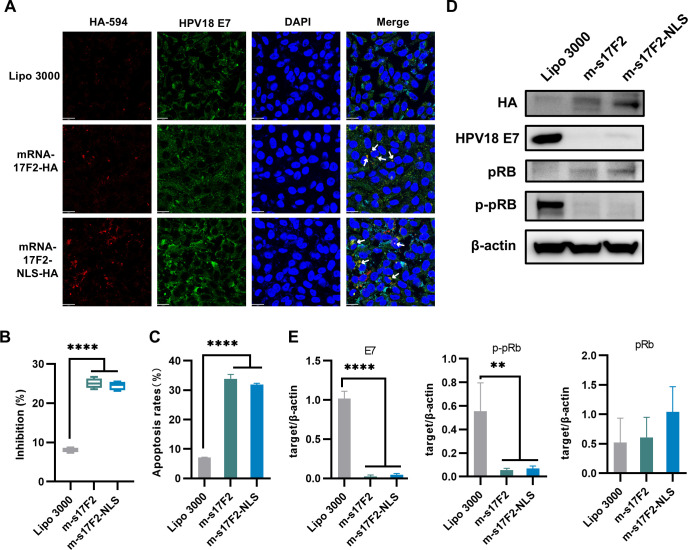
mRNA-s17F2 blocks E7 oncogenic activity, suppressing proliferation and inducing apoptosis in HPV18-transformed HeLa cells. (**A**) Confocal imaging of m-s17F2 (red) colocalized with HPV18 E7 (green) in HPV18 positive HeLa cells. Nuclei are displayed in blue. Scale bar = 20 um. The white arrows indicate the colocalization areas. (**B**) CCK-8 test. Inhibition rate = (1 – experimental group OD450/average value of HeLa) × 100%. (**C**) Flow cytometry assessed apoptosis rates after mRNA or Lipo 3000 transfection. Apoptosis rate is the sum of the rates of late and early apoptotic cells. (**D**) WB analysis of endogenous molecular signaling in HeLa cells post-mRNA transfection. mRNA transfection significantly downregulates the levels of E7 and p-pRb, while upregulating the level of pRb. (**E**) Quantitative analysis of (**D**) using ImageJ. Difference analysis uses one-way ANOVA with the Dunnett’s multiple comparisons test, compared with the control group (PLVX), *n* = 3, *P < 0.05, **P < 0.01, ***P < 0.001, and ****P < 0.0001.

Immunofluorescence analysis performed 24 h post-transfection showed co-localization between E7 protein and scFv-17F2. In the m-s17F2 group, co-localization was primarily observed in the perinuclear region, while in the m-s17F2-NLS group, co-localization occurred both around and within the nucleus, accompanied by evident nucleic acid leakage (Fig. 5A). CCK-8 assays revealed that both mRNA constructs significantly inhibited HeLa cell proliferation (Fig. 5B), and Annexin V-PE/FITC staining confirmed substantial induction of apoptosis (Fig. 5C; Fig. S7B).

WB conducted 48 h post-transfection showed that mRNA-scFv markedly suppressed E7 protein expression while upregulating total pRb and decreasing its phosphorylated (inactive) form. These findings suggest successful intracellular expression of the mRNA constructs, which likely leads to E7 degradation and the functional restoration of pRb-mediated tumor suppression (Fig. 5D and E).

### mRNA-encoded scFv-17F2 suppresses tumor growth in a mouse model of HPV18+ cancer

**Fig 6:**
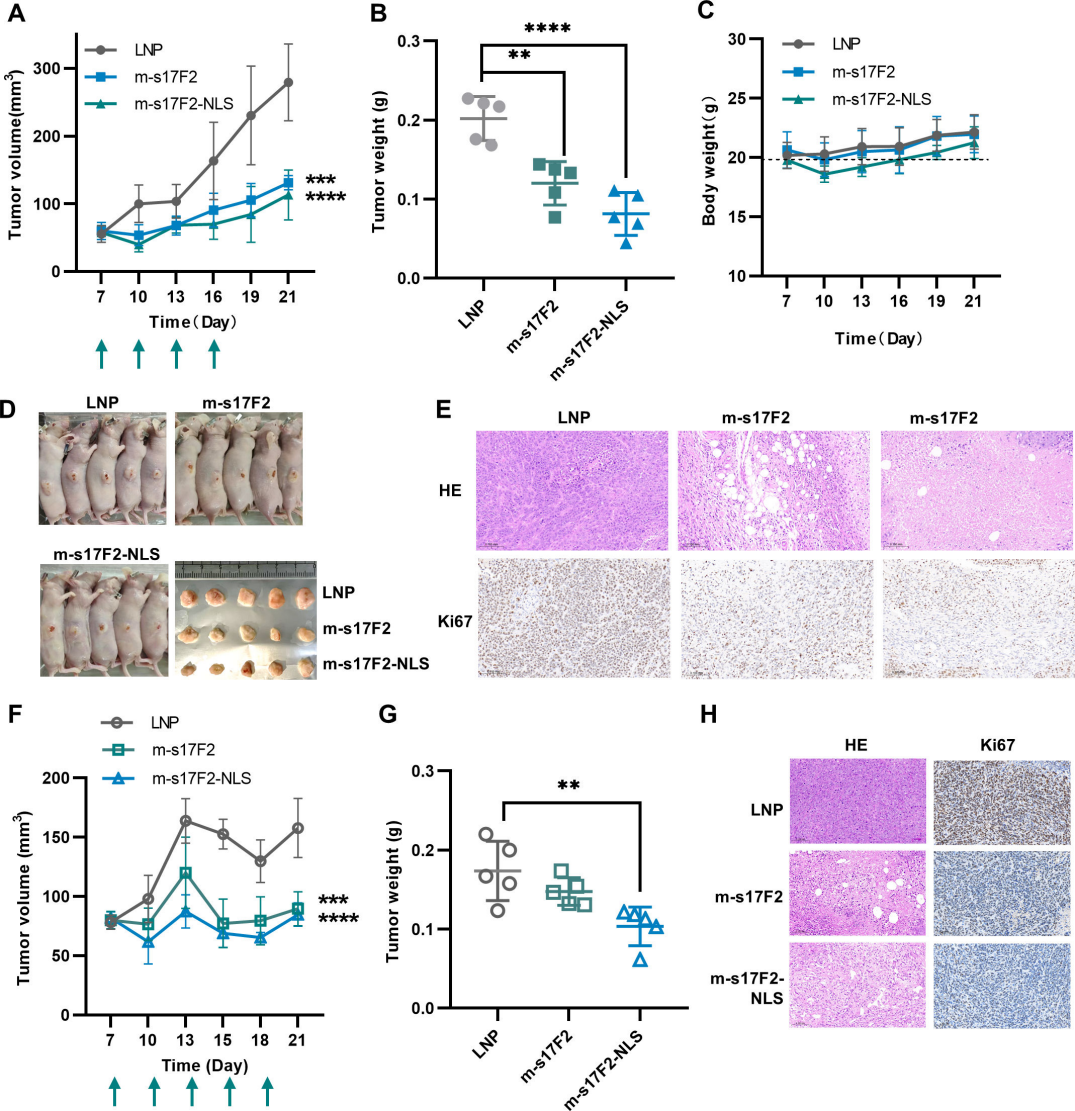
Effectiveness of m-s17F2 against HeLa solid tumors in immunodeficient mice. (**A**) Tumor growth curves in nude mice treated with LNP, m-s17F2, and m-s17F2-NLS. Arrows indicate the mRNA injections. Measurements were taken every 3 days. One-way ANOVA with the Dunnett’s multiple comparisons test, ***P < 0.001 and ****P < 0.0001, data of day 21 compared with the control group: LNP, *n* = 5. (**B**) The tumor weight at the endpoint of treatment. **P < 0.01 and ****P < 0.0001. (**C**) Animal weight change curves during mRNA administration. (**D**) Tumor status of animals at therapeutic endpoints. (**E**) HE staining and Ki67 marker staining of tumor tissues of nude mice. (**F**) Tumor growth curves in NOD-SCID mice treated with LNP, m-s17F2, and m-s17F2-NLS. Arrows indicate the mRNA injections. Measurements were taken every 3 days. ***P < 0.001 and ****P< 0.0001, compared with the control group: LNP, *n* = 5. (**G**) The tumor weight at the endpoint of treatment of NOD-SCID mice, **P < 0.01. (**H**) HE staining and Ki67 marker staining of tumor tissues of NOD-SCID mice.

We next validated the *in vivo* efficacy of two mRNA-scFv constructs using a nude mouse HeLa xenograft model. Lipid nanoparticle (LNP)-encapsulated mRNA appeared as larger, droplet-shaped particles compared to LNPs alone (Fig. S7C). A dosage of 2 mg/kg was administered intratumorally every 3 days for a total of four doses (Fig. 6A). Throughout the treatment, tumor volume and body weight were monitored every 3 days. At the study endpoint, tumors were harvested for weight measurement and histological evaluation.

After a 21-day treatment period, the tumor volumes of the two treatment groups of mice were significantly reduced, and the m-s17F2-NLS group demonstrated superior therapeutic efficacy (Fig. 6A). The same conclusion can also be observed in the photos of mice (Fig. 6D). The tumor tissue weight in the treatment groups was significantly lower than that in the control group (Fig. 6B). These findings demonstrate the notable efficacy of mRNA-scFv. Mouse body weights in all groups returned to baseline levels by the end of treatment (Fig. 6C), and no pathological changes were observed in the major organs, indicating acceptable safety (Fig. S8). Histological analysis (H&E staining) and Ki67 immunohistochemistry revealed extensive vacuolization and disorganized tumor architecture in the mRNA-scFv group, along with a marked decrease in Ki67 expression (Fig. 6E), suggesting effective induction of tumor necrosis.

To further validate these findings, a parallel study was conducted in NOD-SCID mice using a lower mRNA dosage of 1 mg/kg, administered intratumorally every 3 days for a total of five doses. Consistent with earlier results, the mRNA-treated groups showed significant reductions in tumor volume, weight, and Ki67 expression compared to controls. H&E staining again revealed disrupted tumor architecture (Fig. 6F through H). Notably, m-s17F2-NLS maintained superior efficacy across both models.

Collectively, these results demonstrate that mRNA-encoded scFv-17F2 exerts potent antiviral activity by neutralizing the viral oncoprotein E7, effectively suppressing HPV18+ tumor growth. The enhanced efficacy of the m-s17F2-NLS construct underscores its promise as a candidate for further development.

## DISCUSSION

In this study, we first identified six clones with high binding activity and affinity for HPV18 E7 using standard assays, then verified their capacity for *in situ* antigen recognition. These antibodies were subsequently detected using AIA assay to assess their cytotoxicity. This approach ultimately yielded a promising candidate antibody-17F2 for further development. Notably, the method demonstrated high specificity, as no cytotoxic effects were observed in non-targeted cells.

Conventionally, antibody therapeutic potential is evaluated based on binding affinity and epitope recognition. However, our results revealed that high-affinity binding alone is insufficient to predict therapeutic efficacy. The analysis of different antibody binding sites suggests that 17F2 theoretically spans the top of the E7 dimer, thereby obstructing E7’s interaction with cell cycle-related proteins. In contrast, 5C5’s binding footprint is located toward the middle and lower regions of E7, rendering it ineffective in inhibiting E7’s function. The other four antibodies favor side binding with the α3 domain of a single E7 monomer, leading to only limited therapeutic effects. Notably, all antibodies, including 5C5, bind to the loop region of the zinc finger domain, specifically at amino acids 64–71. This implies that the therapeutic efficacy of the antibodies may not depend on the entire zinc finger domain but rather on the groove at the top of the E7 dimer involving α3. Moreover, AF3 predictions indicate that the flexible N-terminus of E7 may fold into this top groove, suggesting that the antibodies’ efficacy might stem from binding to this domain, thereby impeding molecular interactions involving the flexible N-terminus.

This region was further validated as an antiviral target through intracellular expression of scFv, a widely used strategy for evaluating HPV-targeted intracellular antibodies. Our data demonstrated that scFv-17F2 inhibited tumor cell proliferation by co-localizing with E7, downregulating E7 protein levels, and restoring pRb level, which collectively induced apoptosis in HeLa cells. The antitumor effects were further corroborated by significant suppression of tumorigenicity in nude mice.

In the process of screening antibodies targeting HPV cancer proteins, a range of candidate molecules has been identified, including scFv and VHH targeting 16E6 (23, 31, 32, 35) and 16E7 (34, 36). These antibodies target intracellular E6 or E7 and compensate for the biological activity of P53 or pRB, demonstrating significant therapeutic potential. However, antibody drugs themselves exhibit low cellular entry efficiency, which complicates the direct verification of therapeutic effects in animal models. Consequently, most studies use an indirect approach by transducing DNA into cells to investigate cellular proliferation and tumorigenic activity (23, 34). Some studies employed more direct therapeutic methods, such as intratumoral antibody injections (37) or plasmid electroporation for drug delivery (24), demonstrating a certain level of therapeutic efficacy and confirming the effectiveness of the molecules. However, these molecular delivery systems require further enhancement.

To directly assess the antiviral effects of scFv-17F2, we examined the potential of mRNA-delivered scFv antibodies—an ideal format for intracellular protein therapeutics (20). While mRNA-based antibodies have previously been employed against extracellular targets or for blocking viral entry (38–41), their application for intracellular targets remains underexplored. We demonstrated that mRNA-scFvs can be efficiently expressed in cells and exert potent cytotoxic effects. WB confirmed stronger modulation of E7 and pRb signaling pathways compared to lentiviral transduction. *In vivo*, mRNA-scFv constructs significantly inhibited tumor growth in both nude and NOD-SCID mice, even at lower doses. These findings establish mRNA-scFv as a direct and feasible therapeutic approach for HPV-positive tumors, particularly in immunocompromised individuals.

Our research also emphasizes the therapeutic implications of specific epitopes within HPV18 E7. mRNA scFvs designed to target these epitopes have shown significant antitumor efficacy. Notably, none of the six antibodies bound to the α1 region at the base of the E7 dimer, which is a site known to interact with PTPN14. This finding suggests that this epitope may have similar therapeutic potential. Moreover, targeting multiple epitopes could further enhance therapeutic outcomes. Considering the frequent co-infection of different HPV types and the low conservation of oncogenic proteins among these types, patients could be stratified through HPV genotyping, thus allowing for the development of personalized mRNA-scFv cocktails tailored to the tumor’s specific etiology.

In conclusion, our work establishes a functional screening platform for intracellular antibodies, identifies a critical antiviral epitope on the HPV18 E7 oncoprotein, and validates mRNA-encoded scFv as a direct and potent modality to neutralize viral oncoproteins and treat HPV-associated malignancies.

## MATERIALS AND METHODS

### Animals

Male F1 mice were obtained from Hangzhou Ziyuan Laboratory Animal Technology Co. Ltd. Nude mice and NOD-SCID mice were obtained from Shanghai Model Organisms Center, Inc.

### Cell lines, lentivirus production, and cell line infection

HeLa, CT26, and B16F10 cells were procured from the American Type Culture Collection (ATCC). Cells were maintained in DMEM cell-culture medium supplemented with 10% FBS and 1% penicillin/streptomycin. PLVX, s17F2, and s17F2-NLS cells were sorted from HeLa cells following lentiviral-mediated transduction of pLVX-AcGFP-N1, PLVX-s17F2, and PLVX-s17F2-NLS. The cells were maintained in DMEM cell-culture medium supplemented with 10% FBS, 1% penicillin/streptomycin, and 0.1% puromycin. All cell cultures were incubated at 37°C, 5% CO_2_ atmosphere. Monoclonal antibody cells were maintained at 1640 cell-culture medium supplemented with 10% FBS and 1% penicillin/streptomycin.

Variable region sequence of 17F2 was cloned into the mammalian expression plasmid pLVX-AcGFP-N1 (Youbio, no. VT1462). The recombinant lentiviral vectors pLVX-s17F2, pLVX-s17F2-NLS, and packaging vectors (psPAX2 and pMD2.G) were co-transfected into 293 FT cells. The culture supernatants were collected at 48 h after transfection, and the virus-containing cell culture medium from transfected cells was filtered. For virus infection, HeLa cells were seeded into six-well plates and infected with polybrene (Santa Cruz, no. sc-134220). Following 24 h incubation, the medium was changed with puromycin (InvivoGen, no. ant-pr-1) for selection and cultured at 37°C in 5% CO_2_ incubator. After 48 h, the infected cells were passaged regularly in 3–5 days. After 7 days of screening, cells were named PLVX, s17F2, and s17F2-NLS cells.

### Protein expression and purification

#### HPV18E7 and its mutants

The HPV18E7 WT amino sequence obtained from UniProt (P06788). HPV18E7 WT and mutants K67A, E69A, W100A, Q104A, Q105A, and N-terminal were connected with 6× His tag and cloned into the pTO-T7 vector. The plasmid is transformed into *Escherichia coli* BL21 (DE3) strain, and a single colony was overnight inoculated into 3 mL LB medium with 50 μg/mL kanamycin at 37°C. After incubation, 1 mL of inoculated LB medium was transferred to fresh 1 L of LB medium, and 0.4 mM IPTG was added to induce protein expression when the OD600nm of the fresh LB medium reached 0.6; the culture was then incubated at 24°C for an additional 12 h. The cells were harvested and resuspended in lysis buffer (50 mM Tris [pH 7.5], 150 mM NaCl). Suspended cells were sonicated in ice and centrifuged at 25,000 × *g* for 15 min at 4°C. The soluble fraction passed through a 0.22 μm filter was purified by the Ni Sepharose 6 Fast Flow resin (Cytiva, no.17531803), and elution components were collected and purified using Superdex 75 Increase 10/300 GL (Cytiva, no.29148721). Components with different peak times were collected and identified, and the target protein was obtained.

### High-performance size-exclusion chromatography

The homogeneity of HPV18 E7 and mutants was assessed using an Agilent 1200 high-performance liquid chromatography system, with a pre-installed TSK G3000 pwxl column. The system flow rate was 0.5 mL/min, and the detection wavelength was set to 280 nm.

### Monoclonal antibody and antibody Fab production

The stable monoclonal hybridomas were injected into the peritoneal cavity of mice to generate ascites fluid. The monoclonal antibody was purified by MabSelect SuRe (Cytiva, no. 17543802). Fab fragments were generated by digesting IgG with papain (Sigma-Aldrich, no. P4762) and removing IgG and Fc contaminants using MabSelect SuRe.

#### scFv-17F2

The light chain and heavy chain genes of the variable region of antibody 17F2 were linked by 3×GGGS, secreted signaling peptide was added to the N-terminal, and 8× His tag was added to the C-terminal, and the gene was cloned to the PTT5 vector. The correct clones were transformed into DH5α, and single colonies were selected and cultured in 4mL LB medium with 50 μg/mL kanamycin at 37°C. After incubation, 1 mL inoculated LB medium was transferred to fresh 0.5 L LB medium. Endo-Free Maxi Plasmid Kit (Tian gen, no. DP117) was used for plasmid extraction. The obtained plasmid was transfected into 293 F cell, and transient plasmid transfer was performed using the ATM293 system (Zhuhai Kerry, no. K03252, K03125, K30001, K20001, and K40001), and the culture was harvested 6 days after transfection. The culture was centrifuged at 3,000 *g* for 10 min. After filtration with a 0.22 μm filter membrane, the supernatant was purified with Ni Sepharose excel resin (Cytiva, no. 17371203), and the elution components were collected. After concentration, Superdex 75 Increase 10/300 GL (Cytiva, no.29148721) was used for purification. Components with different peak times were collected for identification.

### Enzyme-linked immunosorbent assay

Microtiter plates (96-well) were coated with 100 ng per well of either the E7 or mutants and incubated for 2 h at 37°C. Subsequently, 100 μL of twofold serially diluted antibody was added to each well, and the plates were incubated for an additional h at 37°C. The wells were then washed five times. Next, 100 μL of horseradish peroxidase (HRP)-conjugated goat anti-mouse (Abcam, no. ab97265) at 1:5,000 was added to each well for 1 h at 37°C. After five rinses, 100 μL per well of 3,3,5,5-tetramethylbenzidine substrate solution (Wantai BioPharm, China) was added, followed by incubation at 37°C for 10 min. The reaction was stopped by adding 50 μL of 2 M H_2_SO_4_ to each well, and the OD value was determined at 450 nm with a reference wavelength of 630 nm. The EC50 was calculated using sigmoid trend fitting in GraphPad Prism 8 (GraphPad Software, CA, USA).

For antibody epitope-blocking experiments, monoclonal antibodies (2E8, 5C5, 8A7, 9A5, 14E5, and 17F2) were labeled with HRP, and ELISA was used to determine the concentration of the enzymatic antibody with similar OD450nm response value. The mixed system of enzymatic antibody (100 μL/well) and un-enzymic antibody (5 μg/well excess) was added to 96-well microtiter plates coated with HPV18E7 and incubated at 37°C for 30 min. PBST washes were performed five times, color was developed, and the OD value was determined at 450 nm. The enzymatic antibody with the ED buffer was used as the positive control. The competition rate of antibody was calculated as: competition blocking rate = (1 − OD450 _experiment_/OD450 _positive_) × 100%.

### Surface plasmon resonance

To measure the binding kinetics of mAbs or scFv-17F2 for E7, E7 was immobilized on CM5 chip surface via an amine coupling. PBS-P+ buffer (1×; Cytiva, no. 28995084) was used as the running buffer, serial dilutions of mAbs were injected over immobilized E7 with a flow rate of 30 μL/min for 120 s before dissociation for 300 s. The chip was then regenerated with a 30 s injection of 10 mM glycine pH 1.5. KD values for mAb binding were determined by fitting data to a 1:1 binding isotherm equation using Biacore evaluation software (Cytiva).

To measure binding kinetics of E7 or mutants for antibody 17F2, 17F2 was immobilized on CM5 chip surface via an amine coupling, and serial dilutions of E7 or mutants were injected over immobilized 17F2.

### SDS-PAGE and WB analysis

Cells were washed with cold phosphate-buffered saline (PBS) and then lysed with radioimmunoprecipitation assay buffer (Beyotime, no. P0013B) supplemented with protease inhibitors (Roche, no. 04693132001). Equal amounts of lysates mixed with loading buffer or purified product were separated by SDS-polyacrylamide gel electrophoresis (GenScript; no. M00654) and gel stained with Coomassie Brilliant Blue R-250 (Bio-Rad) for 30 min at room temperature.

For WB, gel was transferred to nitrocellulose membranes. The proteins were then probed with specific primary antibodies followed by HRP-conjugated secondary antibodies. The primary antibodies were monoclonal antibodies (2E8, 5C5, 8A7, 9A5, 14E5, and 17F2) and anti-HPV18E7 (Santa Cruz, no. sc-365035; working concentration, 4 μg/mL), anti-HA-HRP (Abcam, no. ab128131, 1:1,000), anti-β-actin-HRP (ProteinTech, HRP-60008, 1:5,000), anti-Rb (CST, no.9309S, 1:100), anti- phospho-Rb (CST, no.8516S, 1:100). Protein quantification was analyzed using ImageJ.

### Immunofluorescence

Cells were seeded in a six-well plate and cultured overnight. To perform immunostaining, cells were washed with PBS, fixed in 4% paraformaldehyde (Beyotime, no. P0099-500mL) at room temperature, and then permeabilized with Immunostaining Permeabilization Solution with Saponin (Beyotime, no. P0095-500mL), Subsequently, the cells were incubated with the indicated primary antibodies, including monoclonal antibodies (2E8, 5C5, 8A7, 9A5, 14E5, and 17F2) and anti-HPV18E7 (Santa Cruz, no. sc-365035) with 4 μg/mL, anti-HA-Tag mAb (CST, 3724S), at 4°C for 1 h. The cells were washed again with PBS (5% FBS was added for blocking), incubated with secondary antibodies—donkey anti-mouse IgG (H + L) highly cross-adsorbed secondary antibody, Alexa Fluor 488 (Thermo Fisher Scientific, A-21202), donkey anti-goat IgG (H + L) cross-adsorbed secondary antibody, Alexa Fluor 594 (Thermo Fisher Scientific, no. A-11012)—for 1 h in the dark at 4°C, and washed with PBS (5% FBS) again. Nuclei were counterstained with DAPI. Images of immunostained samples were obtained using a confocal fluorescence microscope (Leica TCS SP8 X) with a 63× objective lens.

### Antibody-mediated inhibition of cell proliferative activity

To observe the entry of antibodies, monoclonal antibodies were labeled with Atto550-NHS (Merck Sigma-Aldrich, no. 92835-1MG-F). Antibodies-550 are delivered into HeLa cells using Ab-DeliverIN Transfection Reagent (OZ Biosciences, no. AI20500), specifically, at first, seeding 2 × 10^3^ HeLa cells in each of 96 wells overnight. During transfection, the reagent was mixed with 4 μg or 8 μg antibody, incubated at room temperature, supplemented with DMEM to 20 μL, and added to one well. An equal amount of reagent was mixed with PBS as a negative control, and unrelated IgG mixed reagent was used as a positive control to verify the entry of the antibody. After 48 h or 72 h, CCK-8 reagent (Beyotime, no. C0040) was used to detect the OD450nm value, and the inhibition rate was calculated as follows: Inhibition rate = (1 − the OD450nm _experiment_/the mean value of OD450nm _negative control_) × 100%.

### Crystallization and structure determination of antibody Fab

The HPV18 E7 was mixed with each Fab in a 1:4 molar ratio and incubated at 4°C for overnight. The immune complex was further purified to remove any excess Fab by gel filtration on a TSK gel G3000 pwxl column (Tosoh Bioscience) in 50 mM Tris pH 7.5 with 150mM NaCl. The complex was concentrated to ~10 mg/mL for crystallization.

The crystallization was performed using sitting-drop vapor diffusion in the screening stage and hanging drop in micro-seeding optimization at 20°C. Crystals of the 17F2:E7 complex were grown in 0.2 M ammonium sulfate +26% PEG 4000. Crystals were cryo-protected in reservoir solution supplemented with 30% glycerol at 100 K before collection of the diffraction data. Diffraction data were collected at Shanghai Synchrotron Radiation Facility beamline BL17U1 using a DECTRIS EIGER × 16M Detector. The diffraction data were auto-processed with XDS software by aquarium pipeline (42). The complex structures were determined by the molecular replacement method using Phaser (43). The resulting models were manually built in COOT (44), refined with PHENIX (45), and analyzed with MolProbity (46). In brief, one round of rigid-body refinement was performed after molecular replacement phasing. The refined models were manually modified in COOT; coordinates and individual B factors were refined in reciprocal space. TLS refinement was performed in the later stages with auto-searched TLS groups in PHENIX, which were listed in REMARK 3 sections in the deposited cif files. Data collection and structure refinement statistics are summarized in Table S1. All figures were prepared with ChimeraX (47).

### Antibody sample preparation using the SP-MEGD method

Initially, 200 µg of each purified monoclonal antibody (2E8, 5C5, 8A7, 9A5, 14E5, and 17F2) were added to a lysis buffer composed of 6 M guanidine hydrochloride (Gu. HCl), 20 mM dithiothreitol, and 100 mM Tris at pH 8.5 in a volume/mass ratio of approximately 1:1 (µL buffer: µg protein) . The mixture was denatured and reduced at a temperature of 60°C for a duration of 30 min. Subsequently, it was alkylated with 40 mM iodoacetamide for an additional period of 30 min at room temperature while being protected from light.

The alkylated antibody samples were then transferred into pre-chilled ultrafiltration tubes with a molecular weight cutoff of 10 kDa (Millipore, USA), containing a solution of 0.8 mM urea and 50 mM Tris-HCl (pH 8.0) at 4°C to eliminate contaminants that could potentially inhibit enzymatic digestion. Proteases—including trypsin, chymotrypsin, pepsin, elastase, and Asp-N—were introduced at a weight ratio of 1:20 and incubated at 37°C for 6 h; samples were collected every 2 h during this incubation period.

Following digestion, trichloroacetic acid was added to achieve a final concentration of 10% and allowed to react for an additional 30 min at 37°C to complete the reaction process. The resulting supernatant was desalted using Sep-Pak C18 solid phase extraction columns (Waters), lyophilized, and subsequently stored at −20°C or dissolved in 0.1% formic acid prior to analysis via liquid chromatography-tandem mass spectrometry (LC-MS/MS)(48).

### LC-MS/MS analysis

The digested peptides were analyzed using a Vanquish Neo ultra-performance liquid chromatography system coupled with an Orbitrap Eclipse mass spectrometer. The column utilized was packed with PepMap 100 C18 (75 μm × 50 cm, 2 μm, Thermo Fisher Scientific, USA). The analysis was conducted via reversed-phase chromatography employing a gradient over 70 min from 0% to 35% solvent B (comprising 0.1% formic acid in 80% acetonitrile) at a flow rate of 300 nL/min. Solvent A consisted of a solution of 0.1% formic acid.

Full MS1 scans were performed on the mass spectrometer within the acquisition range of m/z 350–2,000, achieving a resolution of 120,000. MS1 scans were acquired utilizing standard automatic gain control settings and a maximum injection time of 100 ms. Precursor ions underwent fragmentation through stepped high-energy collision dissociation (HCD) as well as electron-transfer HCD, with the HCD fragmentation steps set at percentages of 27% and 35%.

### Structure prediction using AlphaFold 3 and molecular docking analysis

AlphaFold 3 is considered to provide improved accuracy in modeling antibody-antigen complexes compared to previous versions (29). In this study, the web-based AlphaFold 3 service was used to predict the structures of HPV18 E7 dimer and multiple antibody–HPV E7 protein complexes. The amino acid sequences of the antibodies, together with the dimeric sequences of the E7 protein, were used as input, and models were generated using default parameters. The resulting complex structures were then analyzed to evaluate the predicted antigen-antibody interaction interfaces and binding features. The molecular docking experiments were performed using the ZDOCK and RDOCK software packages (49, 50). The top poses from the ZDOCK results were further refined using R dock.

### Antibody epitope mapping

Upload AF3 and the structure obtained from molecular docking to PDBePISA to obtain interface information. Then, merge the information obtained from the two methods and use ChimeraX to plot the footprint map.

### Cell proliferation and colony formation assay analysis

Cells were cultured at a density of 2 × 10^3^ cells/well on 96-well plate. CCK-8 was used to evaluate cell proliferation according to the manufacturer’s instructions. For the colony formation assay, cells were seeded in a 6-well plate at a density of 400 cells/well and incubated for 10 days. Cells were then fixed and stained with Giemsa, and the colonies that had formed in each well were counted by immunoSpot S5 UV Analyzer (CTL).

### Annexin V-FITC/PI staining assay

The apoptotic ratio was determined using Annexin V- FITC/PI apoptosis detection kit (Vazyme, A211-01). A total of 3 × 10^5^ cells were harvested and stained with Annexin V- FITC and PI according to the manufacturer’s instructions. Afterward, the samples were analyzed using LSRFortessa X-20 (BD).

### *In vitro* transcription and transfection of mRNA

The sequence of s17F2 and s17F2-NLS was synthesized between NcoI and XhoI cleavage sites of the vector PUC57. The plasmids were linearized with BspQI and served as the template for mRNA IVT using T7 High Yield RNA Transcription Kit (N¹ -Me-Pseudo UTP; Vazyme, no. DD4202), and cap analogs were added during the transcription. After IVT, add DNase I (NEB, no. M0303L) to digest the template, purify RNA using the lithium chloride precipitation, and identify the product with 1% agarose gel electrophoresis. Transfection of mRNA is performed using Lipofectamine 3000 transfection reagent (Thermo, no. L3000015), following the reagent’s manual.

### Encapsulation of mRNA in LNPs and assessment of mRNA encapsulation efficiency

Mix cationic lipids, polyethylene glycol-lipid conjugates, distearoyl phosphatidylcholine, and cholesterol in anhydrous ethanol to obtain liposomes. Dilute mRNA using a citric acid buffer, then use microfluidics to mix the aqueous phase and lipid phase, and perform ultrafiltration to exchange the buffer with PBS.

The encapsulated mRNA content is determined by comparing the mRNA content after LNP disruption with the content of free mRNA. LNP disruption is achieved using 0.2% Triton X-100 prepared in TE buffer. The nucleic acid content in both samples is assessed using the Quant-iT RiboGreen RNA Quantitation Kit (Thermo, no. R11490). The final mRNA encapsulation efficiency is calculated using the formula: mRNA encapsulation efficiency = (mRNA concentration after emulsification disruption − free mRNA concentration)/initial mRNA concentration × 100%. Calculate the mRNA dosage based on the encapsulation concentration obtained from the measurement.

### Transmission electron microscopy

LNP or mRNA-LNP (10 μL) was pipetted onto carbon-coated copper grids for 10 min, absorbing any residual liquid before adding dropwise 2% phosphotungstic acid (pH 6.4) and incubating the reaction for 5 min. An FEI Tecnai T12 transmission electron microscopy with an accelerating voltage of 120 kV was used to observe and measure LNP or mRNA-LNP morphology.

### Mouse xenograft models *in vivo* tumor treatment

To evaluate 17F2 intrabody expression on tumor growth, 5 × 10^6^ cells with integrated expression of s17F2 or control cells (PLVX) were injected subcutaneously in the flanks of nude mice. The tumor volume was monitored every 3 days, and tumor growth curves were presented.

For mRNA therapy, 1 × 10^7^ HeLa cells were injected subcutaneously into the flanks of nude mice or NOD-SCID mice. When the tumor reached a size of about 80 mm^3^, the mice were arbitrarily assigned to different groups. mRNA-LNP was injected into the tumor, and the injections were repeated every 3 days. Tumors were measured every 3 days. Tumor-bearing mice were sacrificed at day 21 after the first treatment, and tumors were weighed and stained by Ki67 marker and HE.

### Statistical analysis

Lines present in the graphs represent the mean as indicated. One-way analysis of variance (ANOVA) with Dunnett’s multiple comparisons test was applied to analyze the differences. Differences were considered significant when the *P* value was less than 0.05 (*P < 0.05, **P < 0.01, ***P < 0.001, and ****P < 0.0001; ns, not significant). All statistical analyses were performed in the GraphPad Prism 8 software.

## Data Availability

The coordinates and structure factors for 17F2 Fab have been deposited in the Protein Data Bank (accession no. 9UP7).

## SUPPLEMENTAL MATERIAL

Supplemental material mbio.02627-25-s0001.pdf Supplemental figures and table; PDB validation report.

ASM does not own the copyrights to Supplemental Material that may be linked to, or accessed through, an article. The authors have granted ASM a non-exclusive, world-wide license to publish the Supplemental Material files. Please contact the corresponding author directly for reuse.
