# Supplementary material for: An mRNA-encoded scFv antibody targeting the helix-α3 of HPV18 E7 oncoprotein as a novel antiviral strategy
Source: mBio. 2026 Feb 2;17(3):e02627-25. doi: 10.1128/mbio.02627-25 (PMC12977463; doi:10.1128/mbio.02627-25)
Supplement: Supplemental material — Supplemental figures and table; PDB validation report. [file mbio.02627-25-s0001.pdf]

A

Antibody	EC50(ng/mL)
F-7	22.05
2E8	8.58
5C5	8.07
8A7	6.69
9A5	239.3
14E5	30.19
17F2	7.81

B

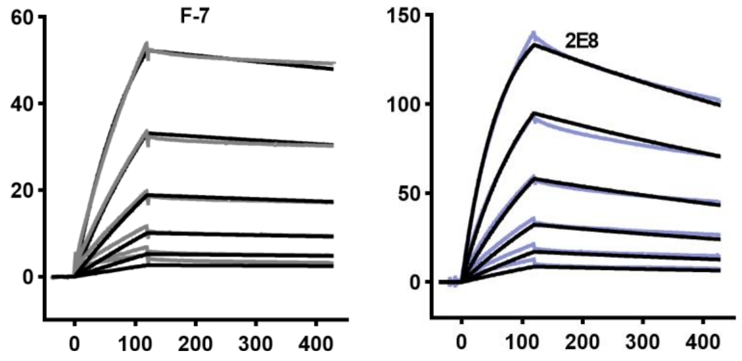

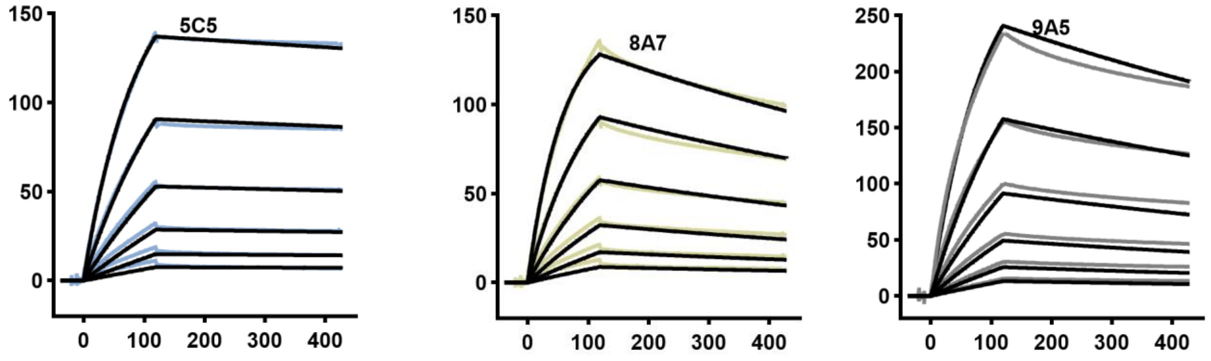

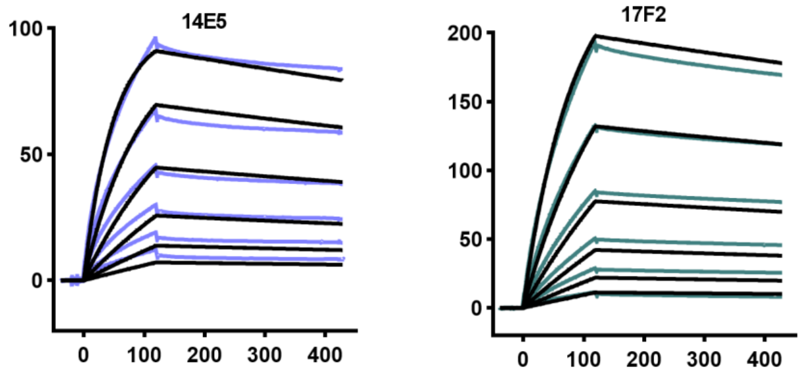

Antibody	KD(M)
F-7	6.07×10^{-9}
2E8	6.17×10^{-9}
5C5	5.66×10^{-9}
8A7	5.54×10^{-9}
9A5	3.45×10^{-9}
14E5	2.23×10^{-9}
17F2	5.66×10^{-9}

Supplemental Figure 1

(A) EC50 of antibodies with HPV18E7.

(B) SPR sensorgrams of antibodies binding to HPV18E7. The table displays the affinity values of antibodies with HPV18 E7.

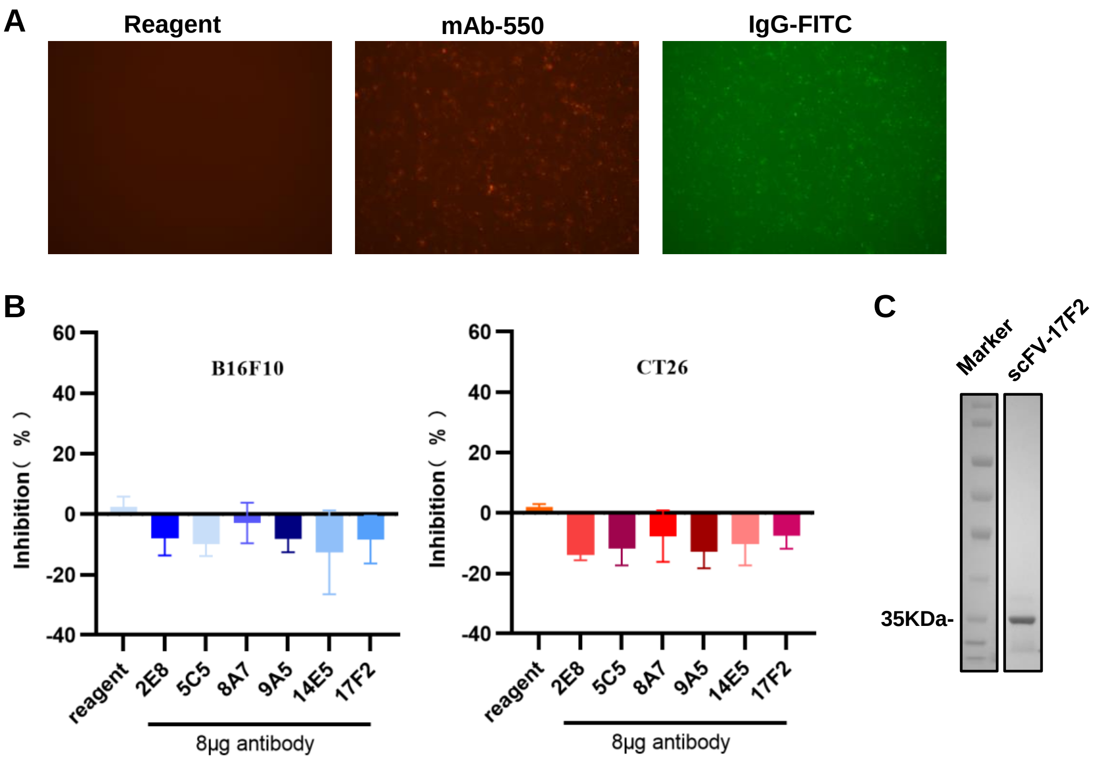

Supplemental Figure 2

(A) Antibody internalization detected by fluorescence microscope.

(B) Antibodies exhibits cell line-specific activity.

(C) purification and SDS-PAGE of scFV-17F2.

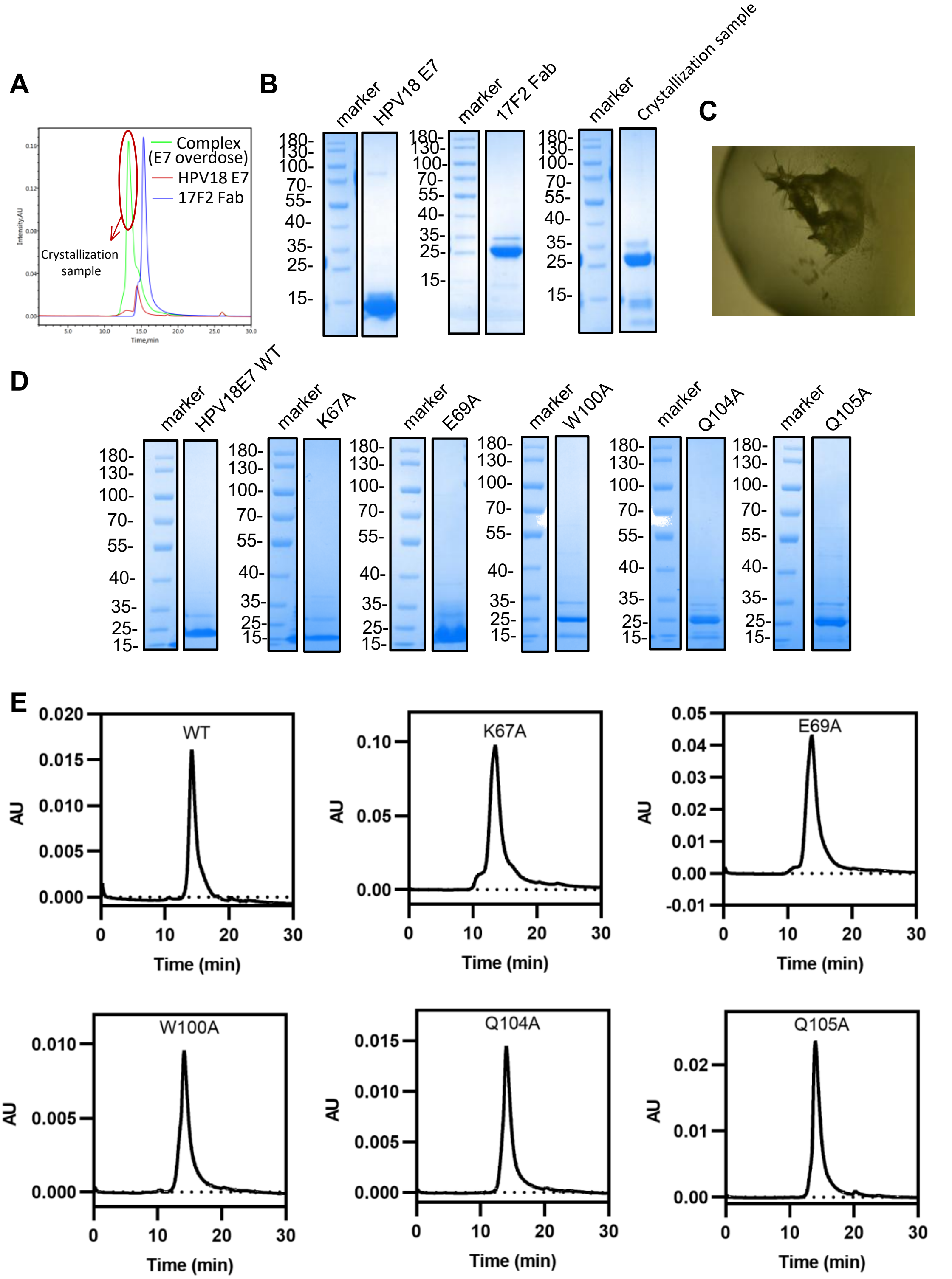

Supplemental Figure 3

(A-C) characterization and crystallization of HPV18E7 and 17F2 Fab complex. A: HPLC B: SDS-PAGE C: crystal.

(D-E) characterization of HPV18E7 mutants. D: SDS-PAGE E: HPLC.

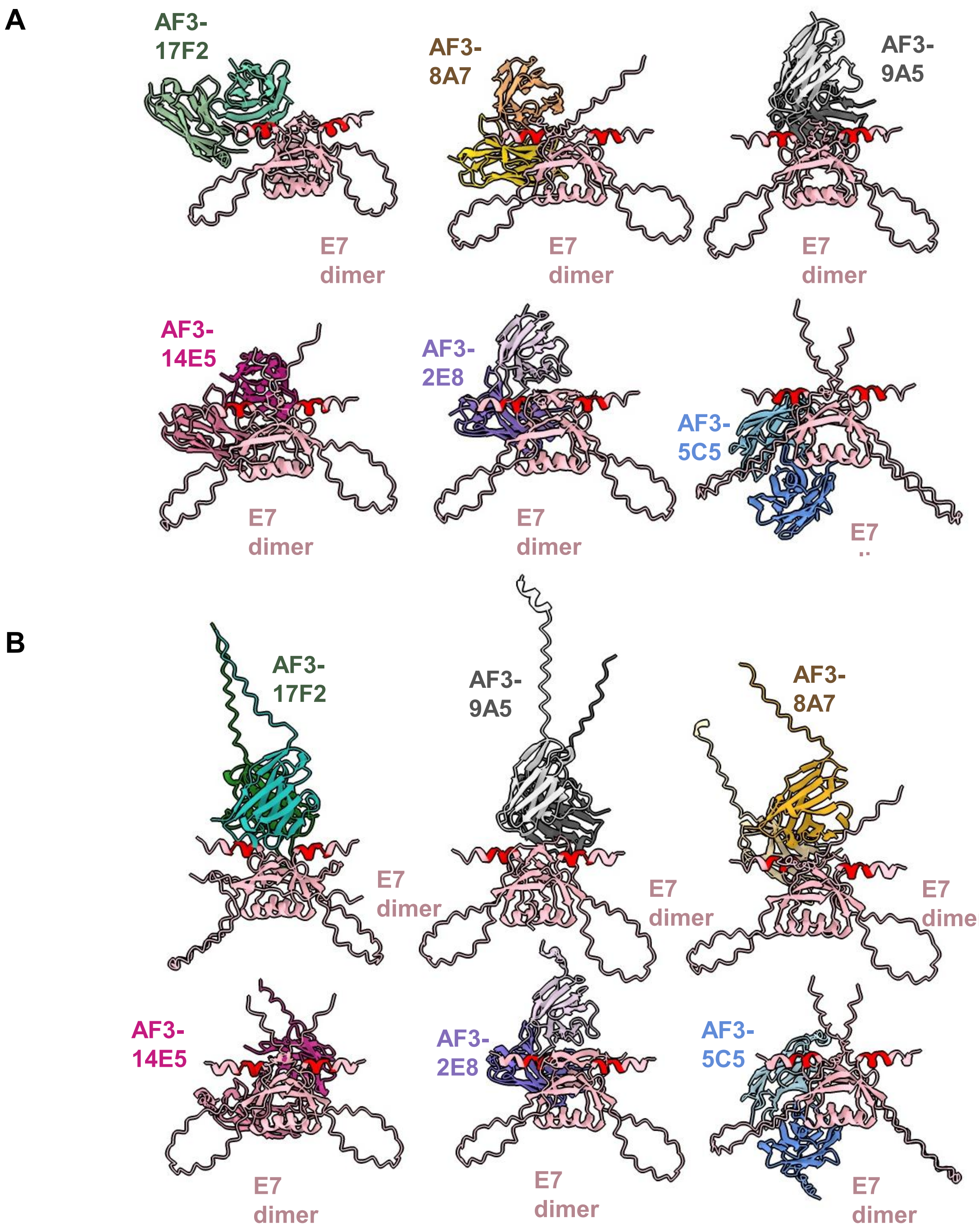

C

molecule	E7 dimer	E7-17F2 Fab complex	E7-9A5 Fab complex	E7-8A7 Fab complex	E7-14E5 Fab complex	E7-2E8 Fab complex	E7-5C5 Fab complex
ipTM value	0.48	0.52	0.53	0.75	0.53	0.53	0.51

Supplemental Figure 4

(A) Molecular Docking of HPV18 E7 Dimer with Six Antibodies

(B) Alphafold3 of HPV18 E7 Dimer with Six Antibodies.

(C) The ipTM value of the structure predicted by AlphaFold3

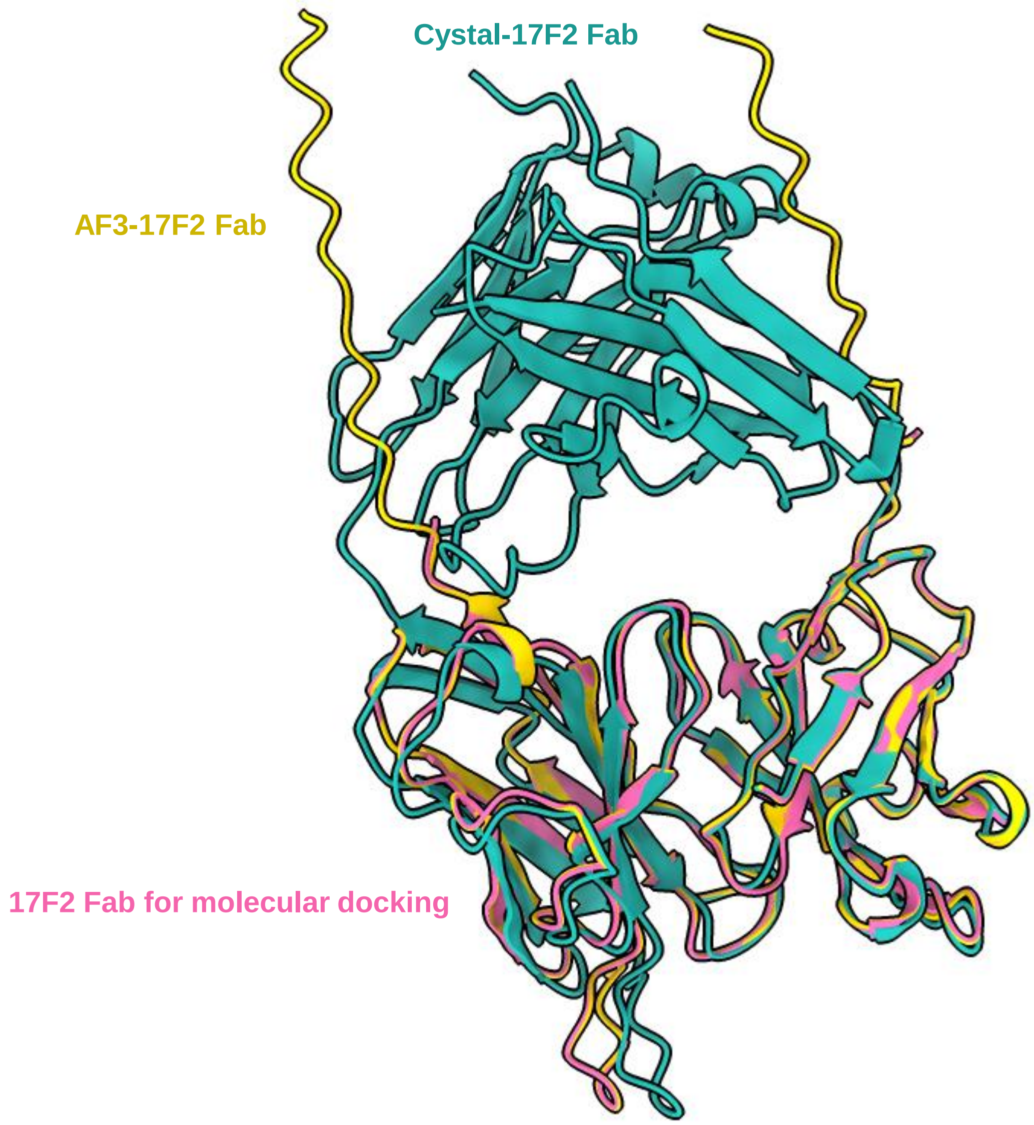

Supplemental Figure 5

17F2 Fab crystal structure comparison with the structure used in the prediction.

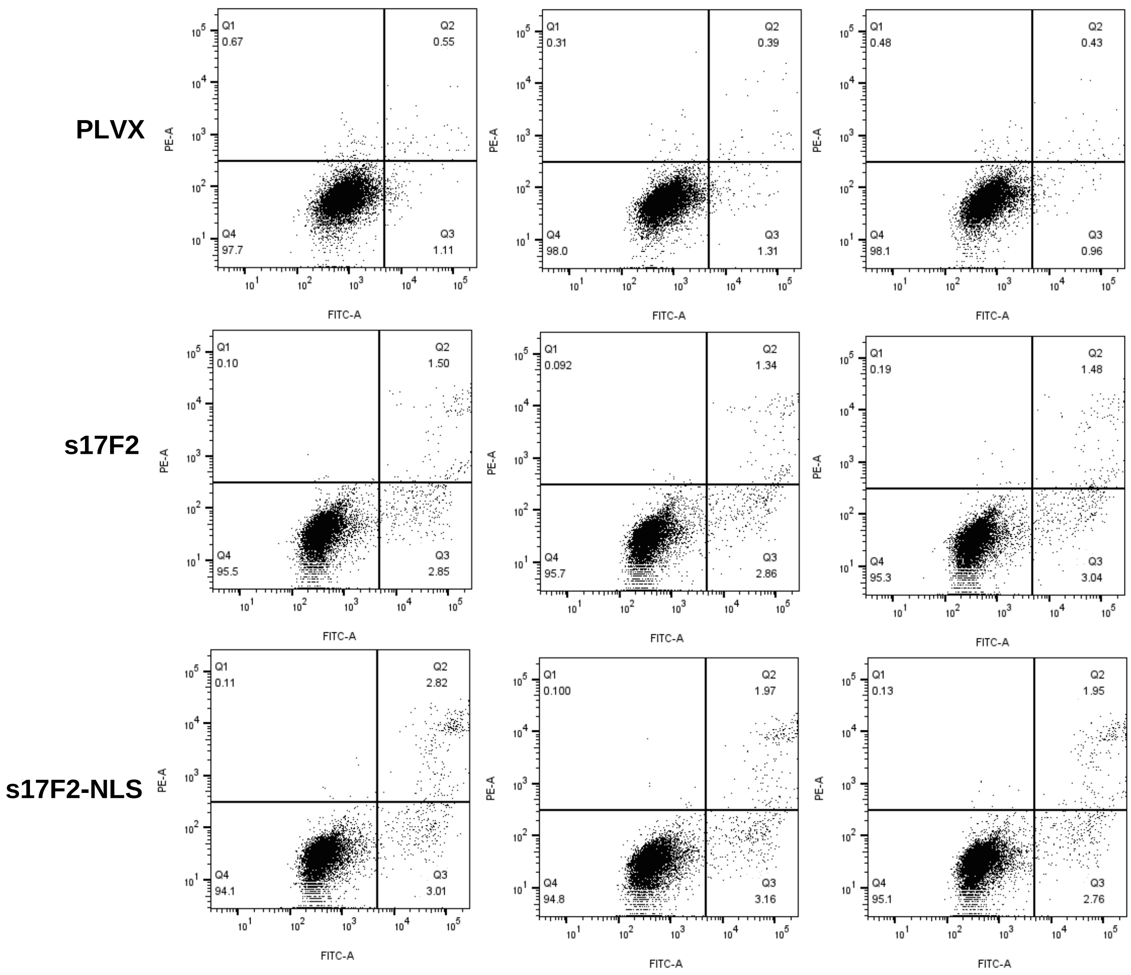

Supplemental Figure 6

Flow cytometry dot plots of Hela cells expressing PLVX, s17F2, and s17F2-NLS.

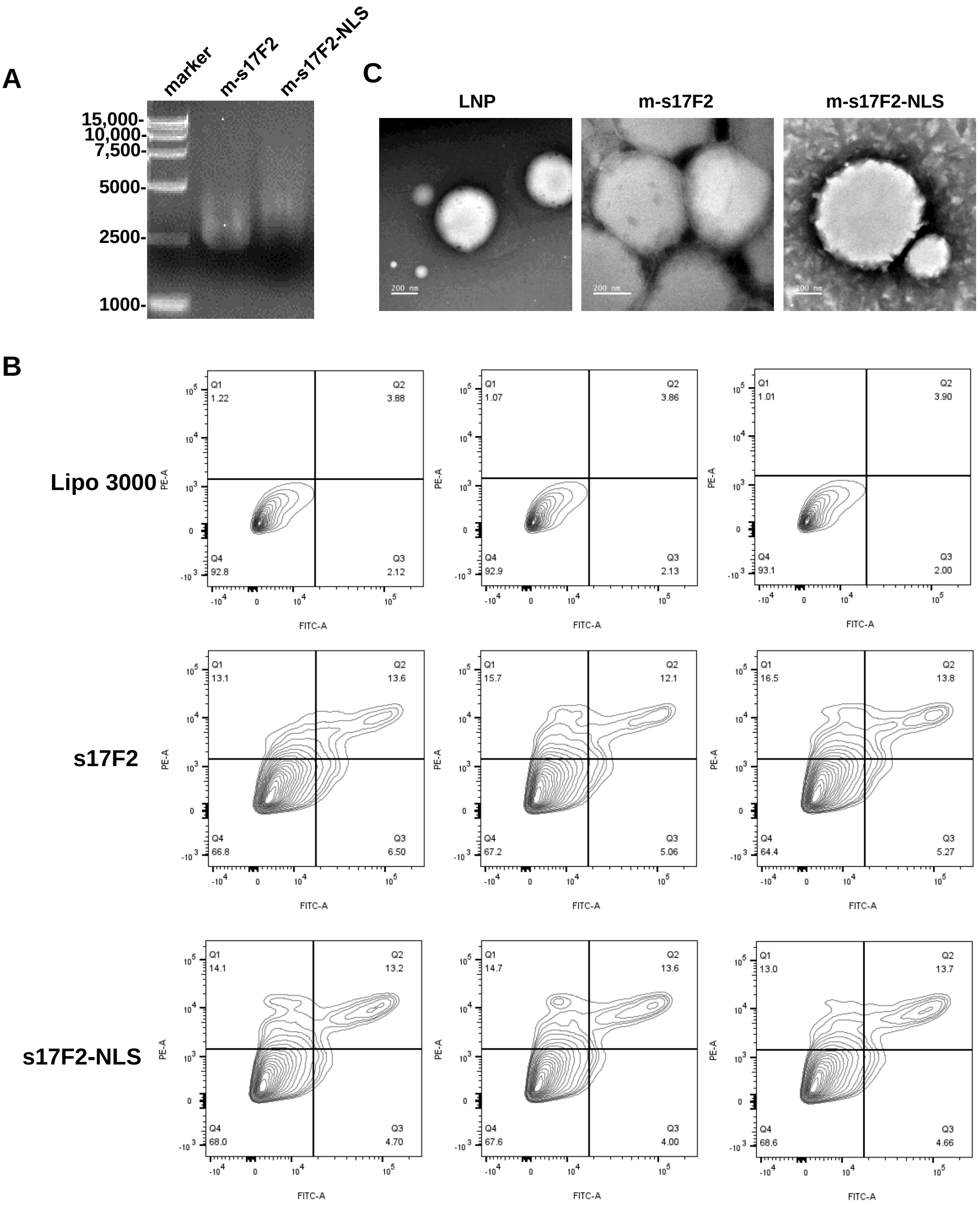

Supplemental Figure 7

(A) mRNA agarose gel electrophoresis characterization.

(B) Morphological characterization of mRNA-LNP by TEM.

(C) Contour plots of flow cytometry apoptosis analysis after mRNA transfection.

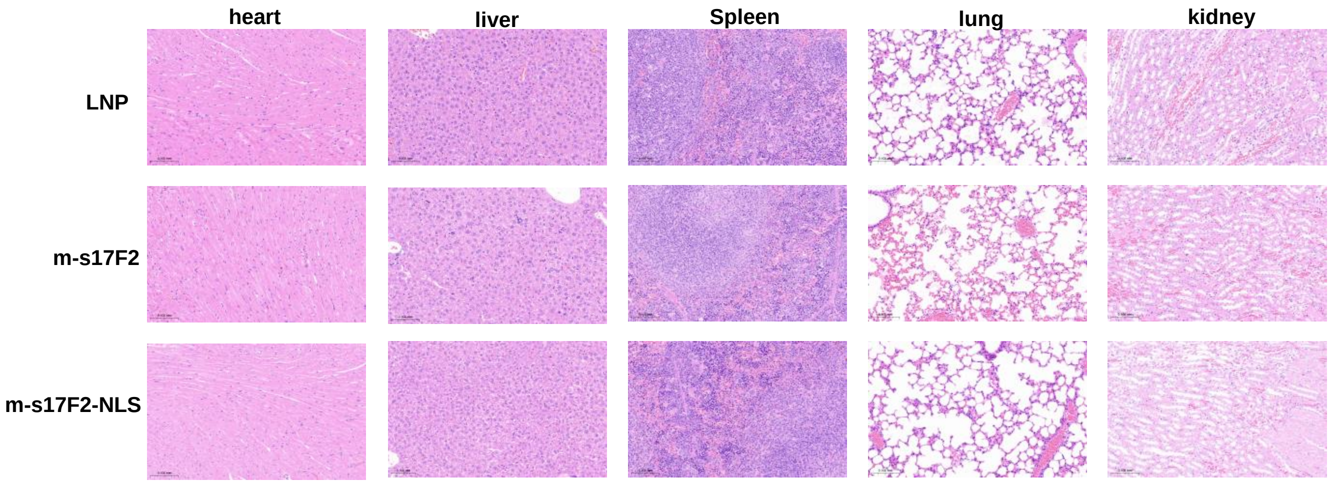

Supplemental Figure 8

HE of organs in nude mice at the treatment endpoint of mRNA-LNP therapy.

Supplemental table 1 Data collection and refinement statistics for 17F2 Fab

Data	Parameters	17F2 Fab	
PDB No.		9UP7	
Data collection			
	Cell parameters (Å, º)	a=67.6,<br>b=54.7,<br>c=124.1	α=90.0,<br>β=102.1,<br>γ=90.0
	Space group	$P2_1$	
	Resolution range [a] (Å)	66.14-2.10 (2.21-2.10)	
	Wavelength (Å)	0.9792	
	Observed hkl (I>σ)	177,759	
	Unique hkl	50,750	
	Redundancy	3.5 (3.2)	
	Completeness (%)	97.3 (95.5)	
	Overall (I/σI)	11.4 (2.4)	
	Rsym [b] (%)	10.9 (80.2)	
Refinement			
	Resolution range (Å)	50.26-2.10	
	Number of Reflections	48,846	
	R_{factor} [c]	21.1	
	R_{free} [d]	25.7	
	RMSD bond lengths (Å)	0.01	
	RMSD bond angles (°)	0.84	
	No. atoms	6,872	
	Protein	6.520	
	Water	354	
	Wilson B-factor (Å^2)	29.1	
	Average B-factors (Å^2)	39.8	
	Protein	39.8	
	Water	40.6	
Ramachandran Plot			
	Favored and allowed region (%)	97.0	
	Generously allowed regions (%)	2.3	
	Disallowed regions (%)	0.7	

[a] Numbers in parentheses refer to the highest resolution shell.
[b] Rsym = Σh Σi|I_1(h) - <I(h)| / Σh Σi I_1(h)
[c] R_{factor} = Σhkl||Fobs| − k|Fcalc||/Σhkl|Fobs|.
[d] R_{free} is calculated using the same equation

Full wwPDB X-ray Structure Validation Report (i)

May 1, 2025 – 01:58 PM JST

PDB ID	:	9UP7 / pdb_00009up7
Title	:	anti-HPV18 E7 monoclonal antibody
Deposited on	:	2025-04-27
Resolution	:	2.10 Å(reported)

This wwPDB validation report is for manuscript review

This is a Full wwPDB X-ray Structure Validation Report.

This report is produced by the wwPDB biocuration pipeline after annotation of the structure.

We welcome your comments at *validation@mail.wwpdb.org*
A user guide is available at
https://www.wwpdb.org/validation/2017/XrayValidationReportHelp
with specific help available everywhere you see the (i) symbol.

The types of validation reports are described at
http://www.wwpdb.org/validation/2017/FAQs#types.

The following versions of software and data (see references (i)) were used in the production of this report:

MolProbity	:	4-5-2 with Phenix2.0rc1
Xtriage (Phenix)	:	2.0rc1
EDS	:	3.0
Percentile statistics	:	20231227.v01 (using entries in the PDB archive December 27th 2023)
CCP4	:	9.0.006 (Gargrove)
Density-Fitness	:	1.0.12
Ideal geometry (proteins)	:	Engh & Huber (2001)
Ideal geometry (DNA, RNA)	:	Parkinson et al. (1996)
Validation Pipeline (wwPDB-VP)	:	2.43.1

For Manuscript Review

1 Overall quality at a glance (i)

The following experimental techniques were used to determine the structure:
X-RAY DIFFRACTION

The reported resolution of this entry is 2.10 Å.

Percentile scores (ranging between 0-100) for global validation metrics of the entry are shown in the following graphic. The table shows the number of entries on which the scores are based.

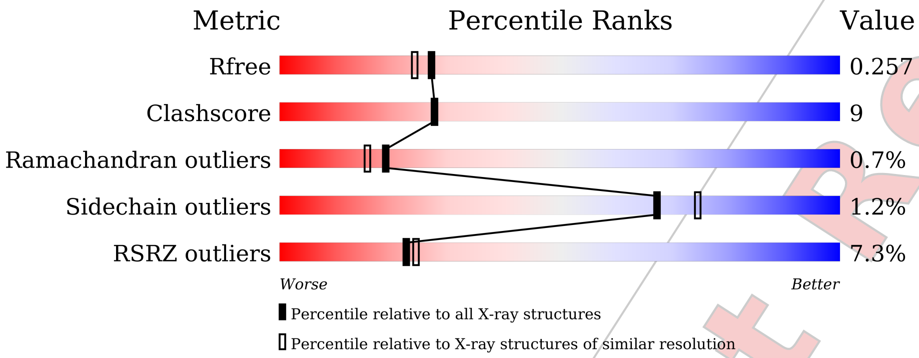

Metric	Whole archive (#Entries)	Similar resolution (#Entries, resolution range(Å))
R_{free}	164625	6234 (2.10-2.10)
Clashscore	180529	6893 (2.10-2.10)
Ramachandran outliers	177936	6839 (2.10-2.10)
Sidechain outliers	177891	6840 (2.10-2.10)
RSRZ outliers	164620	6234 (2.10-2.10)

The table below summarises the geometric issues observed across the polymeric chains and their fit to the electron density. The red, orange, yellow and green segments of the lower bar indicate the fraction of residues that contain outliers for >=3, 2, 1 and 0 types of geometric quality criteria respectively. A grey segment represents the fraction of residues that are not modelled. The numeric value for each fraction is indicated below the corresponding segment, with a dot representing fractions <=5% The upper red bar (where present) indicates the fraction of residues that have poor fit to the electron density. The numeric value is given above the bar.

Mol	Chain	Length	Quality of chain
1	A	214	7% / 86% 10% • •
1	H	214	7% / 78% 17% •
2	B	220	4% / 81% 19%
2	L	220	10% / 81% 18% •

For Manuscript Review

2 Entry composition (i)

There are 3 unique types of molecules in this entry. The entry contains 6874 atoms, of which 0 are hydrogens and 0 are deuteriums.

In the tables below, the ZeroOcc column contains the number of atoms modelled with zero occupancy, the AltConf column contains the number of residues with at least one atom in alternate conformation and the Trace column contains the number of residues modelled with at most 2 atoms.

- Molecule 1 is a protein called Heavy chain of the anti-HPV18 E7 monoclonal antibody 17F2.

Mol	Chain	Residues	Atoms					ZeroOcc	AltConf	Trace
1	A	207	Total 1564	C 989	N 259	O 310	S 6	0	0	0
1	H	205	Total 1556	C 985	N 257	O 308	S 6	0	0	0

- Molecule 2 is a protein called Light chain of the anti-HPV18 E7 monoclonal antibody 17F2.

Mol	Chain	Residues	Atoms					ZeroOcc	AltConf	Trace
2	B	220	Total 1703	C 1061	N 284	O 349	S 9	0	0	0
2	L	219	Total 1697	C 1058	N 283	O 348	S 8	0	0	0

- Molecule 3 is water.

Mol	Chain	Residues	Atoms		ZeroOcc	AltConf
3	A	72	Total 72	O 72	0	0
3	B	120	Total 120	O 120	0	0
3	H	73	Total 73	O 73	0	0
3	L	89	Total 89	O 89	0	0

For Manuscript Review

3 Residue-property plots (i)

These plots are drawn for all protein, RNA, DNA and oligosaccharide chains in the entry. The first graphic for a chain summarises the proportions of the various outlier classes displayed in the second graphic. The second graphic shows the sequence view annotated by issues in geometry and electron density. Residues are color-coded according to the number of geometric quality criteria for which they contain at least one outlier: green = 0, yellow = 1, orange = 2 and red = 3 or more. A red dot above a residue indicates a poor fit to the electron density (RSRZ > 2). Stretches of 2 or more consecutive residues without any outlier are shown as a green connector. Residues present in the sample, but not in the model, are shown in grey.

• Molecule 1: Heavy chain of the anti-HPV18 E7 monoclonal antibody 17F2

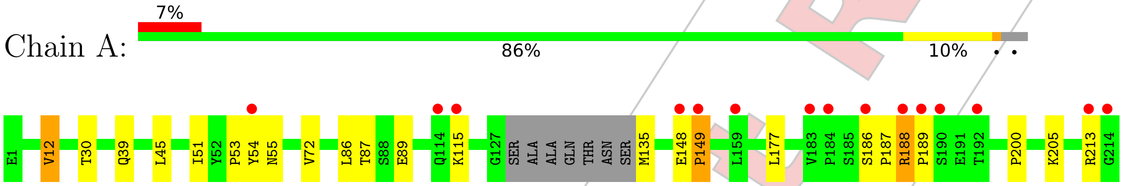

• Molecule 1: Heavy chain of the anti-HPV18 E7 monoclonal antibody 17F2

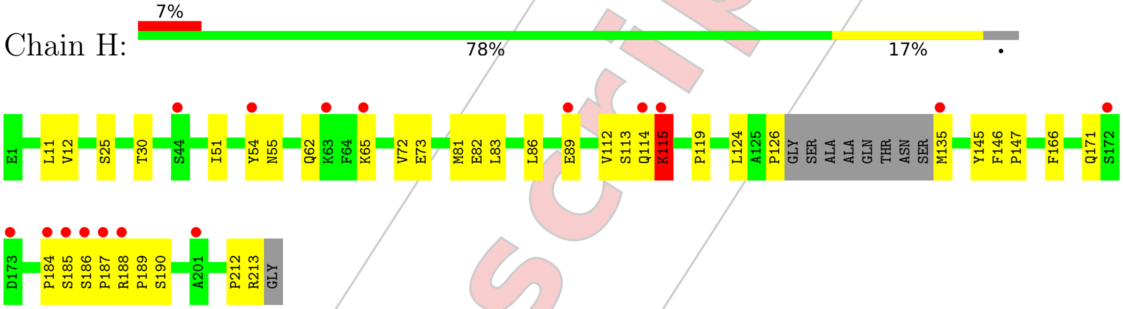

• Molecule 2: Light chain of the anti-HPV18 E7 monoclonal antibody 17F2

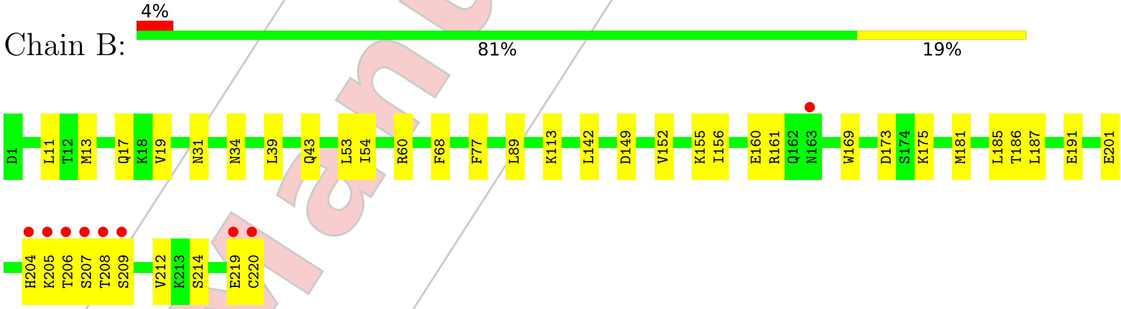

• Molecule 2: Light chain of the anti-HPV18 E7 monoclonal antibody 17F2

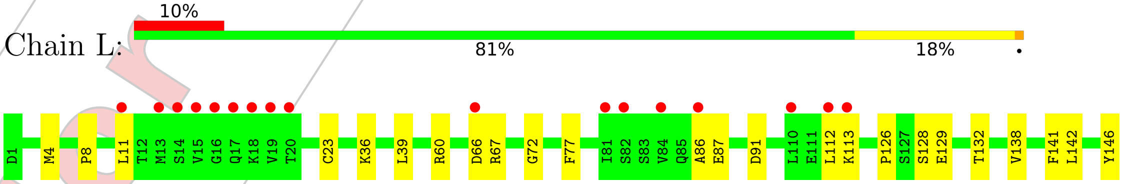

For Manuscript Review

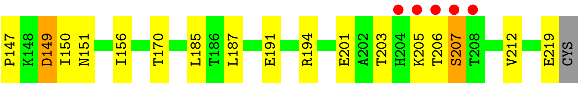

For Manuscript Review

For Manuscript Review

4 Data and refinement statistics (i)

Property	Value	Source
Space group	P 1 21 1	Depositor
Cell constants<br>a, b, c, α, β, γ	67.64Å 54.70Å 124.06Å<br>90.00° 102.11° 90.00°	Depositor
Resolution (Å)	50.26 – 2.10<br>50.26 – 2.10	Depositor<br>EDS
% Data completeness<br>(in resolution range)	93.6 (50.26-2.10)<br>93.7 (50.26-2.10)	Depositor<br>EDS
R_{merge}	0.08	Depositor
R_{sym}	(Not available)	Depositor
$< I/\sigma(I) >$ [1]	2.68 (at 2.10Å)	Xtriage
Refinement program	PHENIX (1.20.1_4487: ???)	Depositor
R, R_{free}	0.211 , 0.257<br>0.214 , 0.257	Depositor<br>DCC
R_{free} test set	50151 reflections (3.95%)	wwPDB-VP
Wilson B-factor (Å^2)	29.2	Xtriage
Anisotropy	0.343	Xtriage
Bulk solvent k_{sol}(e/Å^3), B_{sol}(Å^2)	0.36 , 47.3	EDS
L-test for twinning[2]	$< \lvert L \rvert > = 0.49$, $< L^2 > = 0.32$	Xtriage
Estimated twinning fraction	No twinning to report.	Xtriage
F_o,F_c correlation	0.94	EDS
Total number of atoms	6874	wwPDB-VP
Average B, all atoms (Å^2)	39.0	wwPDB-VP

Xtriage's analysis on translational NCS is as follows: *The largest off-origin peak in the Patterson function is 4.98% of the height of the origin peak. No significant pseudotranslation is detected.*

[1] Intensities estimated from amplitudes.

[2] Theoretical values of $< \lvert L \rvert >$, $< L^2 >$ for acentric reflections are 0.5, 0.333 respectively for untwinned datasets, and 0.375, 0.2 for perfectly twinned datasets.

5 Model quality (i)

5.1 Standard geometry (i)

The Z score for a bond length (or angle) is the number of standard deviations the observed value is removed from the expected value. A bond length (or angle) with $|Z| > 5$ is considered an outlier worth inspection. RMSZ is the root-mean-square of all Z scores of the bond lengths (or angles).

Mol	Chain	Bond lengths		Bond angles	
		RMSZ	#$\|Z\|$ >5	RMSZ	#$\|Z\|$ >5
1	A	0.42	0/1604	0.49	0/2190
1	H	0.41	0/1596	0.47	0/2180
2	B	0.47	0/1739	0.52	0/2358
2	L	0.44	0/1733	0.48	0/2350
All	All	0.44	0/6672	0.49	0/9078

There are no bond length outliers.

There are no bond angle outliers.

There are no chirality outliers.

There are no planarity outliers.

5.2 Too-close contacts (i)

In the following table, the Non-H and H(model) columns list the number of non-hydrogen atoms and hydrogen atoms in the chain respectively. The H(added) column lists the number of hydrogen atoms added and optimized by MolProbity. The Clashes column lists the number of clashes within the asymmetric unit, whereas Symm-Clashes lists symmetry-related clashes.

Mol	Chain	Non-H	H(model)	H(added)	Clashes	Symm-Clashes
1	A	1564	0	1538	16	0
1	H	1556	0	1532	38	0
2	B	1703	0	1645	33	0
2	L	1697	0	1640	29	0
3	A	72	0	0	5	0
3	B	120	0	0	15	2
3	H	73	0	0	18	1
3	L	89	0	0	13	1
All	All	6874	0	6355	115	2

The all-atom clashscore is defined as the number of clashes found per 1000 atoms (including hydrogen atoms). The all-atom clashscore for this structure is 9.

For Manuscript Review

All (115) close contacts within the same asymmetric unit are listed below, sorted by their clash magnitude.

Atom-1	Atom-2	Interatomic distance (Å)	Clash overlap (Å)
1:H:213:ARG:O	3:H:302:HOH:O	1.86	0.93
1:H:73:GLU:OE2	3:H:301:HOH:O	1.85	0.93
1:H:62:GLN:O	3:H:303:HOH:O	1.90	0.89
2:B:214:SER:OG	3:B:301:HOH:O	1.91	0.87
2:L:86:ALA:N	3:L:302:HOH:O	2.07	0.87
2:B:220:CYS:SG	3:B:404:HOH:O	2.31	0.86
1:H:185:SER:N	3:H:304:HOH:O	2.02	0.84
2:B:113:LYS:O	3:B:302:HOH:O	1.97	0.83
2:B:208:THR:O	3:B:304:HOH:O	2.02	0.76
1:A:135:MET:N	3:A:301:HOH:O	2.18	0.75
2:B:160:GLU:OE2	3:B:305:HOH:O	2.05	0.74
1:H:114:GLN:HA	3:H:329:HOH:O	1.87	0.73
2:L:219:GLU:HB2	3:L:359:HOH:O	1.90	0.71
1:H:65:LYS:HB2	3:H:303:HOH:O	1.92	0.70
2:L:67:ARG:NH2	2:L:87:GLU:OE2	2.27	0.66
1:H:185:SER:CB	3:H:304:HOH:O	2.43	0.66
1:H:185:SER:HB3	3:H:304:HOH:O	1.98	0.64
1:H:135:MET:N	3:H:311:HOH:O	2.31	0.64
2:B:205:LYS:HG2	3:B:307:HOH:O	2.00	0.61
1:A:213:ARG:NH2	3:A:302:HOH:O	2.32	0.61
2:L:72:GLY:O	3:L:304:HOH:O	2.16	0.61
2:L:113:LYS:NZ	3:L:308:HOH:O	2.29	0.61
2:B:186:THR:OG1	3:B:306:HOH:O	2.15	0.61
2:L:8:PRO:HG2	2:L:11:LEU:HG	1.84	0.59
2:B:43:GLN:HB2	2:B:53:LEU:HD11	1.85	0.59
2:L:187:LEU:HB3	2:L:191:GLU:HG3	1.86	0.58
2:L:128:SER:O	2:L:132:THR:HG23	2.04	0.57
1:A:148:GLU:O	1:A:149:PRO:C	2.45	0.57
1:H:11:LEU:HB2	1:H:147:PRO:HG3	1.87	0.57
1:H:135:MET:N	3:H:304:HOH:O	2.38	0.56
2:B:152:VAL:HG23	2:B:181:MET:HE1	1.87	0.56
1:H:65:LYS:CB	3:H:303:HOH:O	2.51	0.56
1:A:51:ILE:HD13	1:A:72:VAL:HG13	1.89	0.55
1:H:62:GLN:C	3:H:303:HOH:O	2.41	0.55
2:B:149:ASP:O	3:B:307:HOH:O	2.18	0.54
1:H:115:LYS:NZ	3:H:305:HOH:O	2.10	0.54
2:B:39:LEU:HD22	2:B:77:PHE:CG	2.43	0.54
2:L:129:GLU:OE2	3:L:305:HOH:O	2.18	0.54
2:B:60:ARG:NH1	2:B:68:PHE:O	2.39	0.52
1:H:81:MET:HE3	1:H:83:LEU:HD21	1.91	0.52

Continued on next page...

For Manuscript Review

Continued from previous page...

Atom-1	Atom-2	Interatomic distance (Å)	Clash overlap (Å)
2:B:204:HIS:HD2	2:B:206:THR:HG22	1.74	0.52
2:L:194:ARG:C	3:L:311:HOH:O	2.52	0.51
1:A:213:ARG:CZ	3:A:302:HOH:O	2.58	0.51
2:L:151:ASN:HB2	2:L:203:THR:OG1	2.11	0.51
1:H:188:ARG:HD2	1:H:189:PRO:HA	1.92	0.51
1:H:54:TYR:O	1:H:55:ASN:CG	2.54	0.51
2:L:149:ASP:N	2:L:149:ASP:OD1	2.44	0.50
1:A:54:TYR:O	1:A:55:ASN:CG	2.55	0.50
1:H:212:PRO:HA	3:H:308:HOH:O	2.11	0.50
1:H:25:SER:OG	3:H:306:HOH:O	2.19	0.50
2:L:201:GLU:HG2	2:L:212:VAL:HG12	1.94	0.50
1:H:126:PRO:O	1:H:213:ARG:NE	2.45	0.50
2:L:86:ALA:HA	2:L:112:LEU:HD13	1.94	0.50
2:B:161:ARG:HD3	3:L:366:HOH:O	2.12	0.49
2:L:206:THR:O	2:L:207:SER:CB	2.61	0.49
2:B:205:LYS:N	3:B:313:HOH:O	2.37	0.49
2:B:207:SER:CB	3:B:308:HOH:O	2.61	0.49
1:A:213:ARG:NH1	3:A:302:HOH:O	2.45	0.49
2:B:209:SER:HA	3:B:304:HOH:O	2.13	0.49
1:H:171:GLN:NE2	3:H:310:HOH:O	2.30	0.48
1:H:119:PRO:HB3	1:H:145:TYR:HB3	1.96	0.48
2:L:219:GLU:CB	3:L:359:HOH:O	2.56	0.48
1:A:188:ARG:HD2	1:A:189:PRO:HA	1.95	0.48
1:A:200:PRO:HG2	3:A:304:HOH:O	2.13	0.47
2:B:169:TRP:CZ2	2:B:181:MET:HE2	2.49	0.47
2:B:207:SER:OG	3:B:308:HOH:O	2.20	0.47
2:L:66:ASP:OD2	3:L:306:HOH:O	2.20	0.47
2:B:54:ILE:HD13	2:B:60:ARG:HA	1.95	0.47
1:H:114:GLN:O	1:H:115:LYS:HB3	2.14	0.47
1:H:89:GLU:OE2	3:H:307:HOH:O	2.20	0.47
1:A:39:GLN:HB2	1:A:45:LEU:HD23	1.97	0.46
2:B:11:LEU:HD21	2:B:13:MET:HE2	1.98	0.46
1:H:186:SER:O	1:H:190:SER:OG	2.23	0.46
2:B:142:LEU:HD21	2:B:152:VAL:HG22	1.96	0.46
2:L:39:LEU:HD22	2:L:77:PHE:CG	2.50	0.46
1:A:12:VAL:HG21	1:A:86:LEU:CD1	2.46	0.46
2:B:156:ILE:HD11	2:B:185:LEU:HD21	1.98	0.46
2:B:31:ASN:HB3	2:B:34:ASN:OD1	2.16	0.46
1:H:55:ASN:OD1	1:H:55:ASN:C	2.58	0.46
1:H:83:LEU:HD22	1:H:83:LEU:N	2.30	0.46
1:H:114:GLN:O	1:H:115:LYS:CB	2.63	0.46

Continued on next page...

For Manuscript Review

Continued from previous page...

Atom-1	Atom-2	Interatomic distance (Å)	Clash overlap (Å)
2:L:156:ILE:HD11	2:L:185:LEU:HD21	1.97	0.46
1:H:51:ILE:HD13	1:H:72:VAL:HG13	1.98	0.45
2:B:155:LYS:NZ	3:B:312:HOH:O	2.37	0.45
1:A:87:THR:OG1	1:A:89:GLU:HG2	2.17	0.45
1:A:55:ASN:OD1	1:A:55:ASN:C	2.59	0.45
2:B:17:GLN:NE2	3:B:318:HOH:O	2.50	0.44
1:H:186:SER:N	1:H:187:PRO:HD2	2.32	0.44
2:B:207:SER:HB2	3:B:308:HOH:O	2.17	0.44
2:L:141:PHE:C	2:L:142:LEU:HD12	2.42	0.44
1:H:62:GLN:HA	3:H:303:HOH:O	2.17	0.44
2:L:146:TYR:CG	2:L:147:PRO:HA	2.53	0.44
2:L:86:ALA:HA	2:L:112:LEU:CD1	2.47	0.44
1:H:83:LEU:HB3	1:H:86:LEU:HD21	2.00	0.43
2:B:204:HIS:CD2	2:B:206:THR:HG22	2.51	0.43
1:H:166:PHE:CD2	2:L:170:THR:HG23	2.54	0.43
2:B:201:GLU:HG2	2:B:212:VAL:HG12	1.99	0.43
2:B:89:LEU:C	2:B:89:LEU:HD13	2.43	0.43
2:L:194:ARG:HA	3:L:311:HOH:O	2.18	0.43
2:L:4:MET:HE3	2:L:23:CYS:SG	2.59	0.43
1:H:124:LEU:HD22	3:L:327:HOH:O	2.17	0.43
2:B:11:LEU:HD11	2:B:19:VAL:HG13	2.01	0.42
2:B:187:LEU:HB3	2:B:191:GLU:HG3	2.01	0.42
2:L:206:THR:O	2:L:207:SER:HB3	2.19	0.42
1:A:177:LEU:C	1:A:177:LEU:HD12	2.44	0.42
2:L:91:ASP:OD2	3:L:307:HOH:O	2.22	0.42
1:H:30:THR:HB	1:H:54:TYR:HB2	2.02	0.42
2:L:205:LYS:NZ	3:L:316:HOH:O	2.52	0.41
1:A:186:SER:N	1:A:187:PRO:HD2	2.35	0.41
1:H:115:LYS:O	1:H:146:PHE:HD2	2.04	0.41
2:B:173:ASP:OD2	2:B:175:LYS:HB3	2.21	0.41
1:H:184:PRO:HB2	1:H:187:PRO:HD2	2.03	0.40
1:H:12:VAL:O	1:H:112:VAL:HA	2.22	0.40
1:A:30:THR:HA	1:A:53:PRO:HB2	2.03	0.40
2:L:126:PRO:HD3	2:L:138:VAL:HG22	2.02	0.40

All (2) symmetry-related close contacts are listed below. The label for Atom-2 includes the symmetry operator and encoded unit-cell translations to be applied.

Atom-1	Atom-2	Interatomic distance (Å)	Clash overlap (Å)
3:B:404:HOH:O	3:L:380:HOH:O[2_554]	1.80	0.40
3:B:346:HOH:O	3:H:342:HOH:O[2_544]	2.07	0.13

For Manuscript Review

5.3 Torsion angles (i)

5.3.1 Protein backbone (i)

In the following table, the Percentiles column shows the percent Ramachandran outliers of the chain as a percentile score with respect to all X-ray entries followed by that with respect to entries of similar resolution.

The Analysed column shows the number of residues for which the backbone conformation was analysed, and the total number of residues.

Mol	Chain	Analysed	Favoured	Allowed	Outliers	Percentiles	
1	A	203/214 (95%)	194 (96%)	7 (3%)	2 (1%)	13	9
1	H	201/214 (94%)	194 (96%)	5 (2%)	2 (1%)	13	9
2	B	218/220 (99%)	213 (98%)	4 (2%)	1 (0%)	25	23
2	L	217/220 (99%)	213 (98%)	3 (1%)	1 (0%)	25	23
All	All	839/868 (97%)	814 (97%)	19 (2%)	6 (1%)	19	16

All (6) Ramachandran outliers are listed below:

Mol	Chain	Res	Type
1	A	115	LYS
1	H	115	LYS
2	L	207	SER
2	B	219	GLU
1	A	149	PRO
1	H	113	SER

5.3.2 Protein sidechains (i)

In the following table, the Percentiles column shows the percent sidechain outliers of the chain as a percentile score with respect to all X-ray entries followed by that with respect to entries of similar resolution.

The Analysed column shows the number of residues for which the sidechain conformation was analysed, and the total number of residues.

Mol	Chain	Analysed	Rotameric	Outliers	Percentiles	
1	A	180/185 (97%)	177 (98%)	3 (2%)	56	63
1	H	180/185 (97%)	178 (99%)	2 (1%)	70	77
2	B	198/198 (100%)	198 (100%)	0	100	100
2	L	197/198 (100%)	193 (98%)	4 (2%)	50	57

Continued on next page...

For Manuscript Review

Continued from previous page...

Mol	Chain	Analysed	Rotameric	Outliers	Percentiles	
All	All	755/766 (99%)	746 (99%)	9 (1%)	67	74

All (9) residues with a non-rotameric sidechain are listed below:

Mol	Chain	Res	Type
1	A	12	VAL
1	A	188	ARG
1	A	205	LYS
1	H	82	GLU
1	H	115	LYS
2	L	36	LYS
2	L	60	ARG
2	L	149	ASP
2	L	150	ILE

Sometimes sidechains can be flipped to improve hydrogen bonding and reduce clashes. All (5) such sidechains are listed below:

Mol	Chain	Res	Type
1	A	33	ASN
1	A	171	GLN
2	B	85	GLN
1	H	3	GLN
2	L	48	GLN

5.3.3 RNA ⓘ

There are no RNA molecules in this entry.

5.4 Non-standard residues in protein, DNA, RNA chains ⓘ

There are no non-standard protein/DNA/RNA residues in this entry.

5.5 Carbohydrates ⓘ

There are no oligosaccharides in this entry.

For Manuscript Review

5.6 Ligand geometry (i)

There are no ligands in this entry.

5.7 Other polymers (i)

There are no such residues in this entry.

5.8 Polymer linkage issues (i)

There are no chain breaks in this entry.

For Manuscript Review

6 Fit of model and data (i)

6.1 Protein, DNA and RNA chains (i)

In the following table, the column labelled '#RSRZ> 2' contains the number (and percentage) of RSRZ outliers, followed by percent RSRZ outliers for the chain as percentile scores relative to all X-ray entries and entries of similar resolution. The OWAB column contains the minimum, median, 95th percentile and maximum values of the occupancy-weighted average B-factor per residue. The column labelled 'Q< 0.9' lists the number of (and percentage) of residues with an average occupancy less than 0.9.

Mol	Chain	Analysed	<RSRZ>	#RSRZ>2			OWAB(Å^2)	Q<0.9
1	A	207/214 (96%)	0.48	15 (7%)	23	25	23, 37, 56, 71	0
1	H	205/214 (95%)	0.61	16 (7%)	20	22	22, 42, 64, 76	0
2	B	220/220 (100%)	0.11	9 (4%)	42	44	16, 33, 48, 95	0
2	L	219/220 (99%)	0.55	22 (10%)	14	15	19, 40, 88, 124	0
All	All	851/868 (98%)	0.43	62 (7%)	22	24	16, 37, 65, 124	0

All (62) RSRZ outliers are listed below:

Mol	Chain	Res	Type	RSRZ
2	B	207	SER	5.7
2	B	208	THR	5.1
2	L	15	VAL	4.9
2	L	207	SER	4.6
2	B	206	THR	4.4
2	L	204	HIS	4.1
2	L	82	SER	4.1
1	H	54	TYR	4.1
1	A	213	ARG	3.8
2	L	206	THR	3.7
2	L	205	LYS	3.6
2	L	84	VAL	3.6
2	L	13	MET	3.5
2	L	208	THR	3.4
2	L	112	LEU	3.3
1	H	115	LYS	3.3
1	A	214	GLY	3.3
1	H	135	MET	3.2
2	L	14	SER	3.2
1	A	54	TYR	3.1
1	A	115	LYS	3.1

Continued on next page...

For Manuscript Review

Continued from previous page...

Mol	Chain	Res	Type	RSRZ
2	L	16	GLY	3.1
1	A	183	VAL	3.0
1	H	172	SER	2.9
1	H	184	PRO	2.8
2	B	204	HIS	2.8
1	A	149	PRO	2.8
1	A	190	SER	2.8
2	L	18	LYS	2.7
1	A	184	PRO	2.7
2	L	11	LEU	2.7
1	H	114	GLN	2.7
2	L	113	LYS	2.7
1	A	114	GLN	2.7
2	L	17	GLN	2.6
1	H	63	LYS	2.6
2	L	81	ILE	2.6
1	H	187	PRO	2.6
2	B	220	CYS	2.6
1	H	89	GLU	2.5
1	A	159	LEU	2.5
1	H	188	ARG	2.5
1	H	65	LYS	2.4
2	L	19	VAL	2.4
1	H	186	SER	2.4
2	B	205	LYS	2.4
2	B	209	SER	2.4
2	B	219	GLU	2.4
1	H	201	ALA	2.4
1	A	192	THR	2.4
1	A	189	PRO	2.3
1	H	44	SER	2.3
1	A	188	ARG	2.2
2	L	66	ASP	2.2
1	A	186	SER	2.2
1	H	173	ASP	2.2
2	B	163	ASN	2.2
2	L	110	LEU	2.1
2	L	20	THR	2.1
2	L	86	ALA	2.0
1	A	148	GLU	2.0
1	H	185	SER	2.0

For Manuscript Review

6.2 Non-standard residues in protein, DNA, RNA chains (i)

There are no non-standard protein/DNA/RNA residues in this entry.

6.3 Carbohydrates (i)

There are no monosaccharides in this entry.

6.4 Ligands (i)

There are no ligands in this entry.

6.5 Other polymers (i)

There are no such residues in this entry.

For Manuscript Review
